# Inflammation and epithelial repair predict mortality, hospital readmission and growth recovery in complicated severe acute malnutrition

**DOI:** 10.1126/scitranslmed.adh0673

**Published:** 2024-02-28

**Authors:** Jonathan P Sturgeon, Joice Tome, Cherlynn Dumbura, Florence D Majo, Deophine Ngosa, Kuda Mutasa, Kanekwa Zyambo, Ellen Besa, Kanta Chandwe, Chanda Kapoma, Benjamin Mwapenya, Kusum J Nathoo, Claire D Bourke, Robert Ntozini, Bernard Chasekwa, Melanie Smuk, Mutsa Bwakura-Dangarembizi, Beatrice Amadi, Paul Kelly, Andrew J Prendergast

**Affiliations:** 1 Zvitambo Institute for Maternal and Child Health Research, Harare, Zimbabwe; 2 Blizard Institute, Queen Mary University of London, London, E1 2AT, UK; 3 Tropical Gastroenterology and Nutrition Group, University of Zambia, Lusaka, Zambia; 4 Faculty of Medicine and Health Sciences, University of Zimbabwe, Harare, Zimbabwe

## Abstract

Severe acute malnutrition (SAM) is the most high-risk form of undernutrition, particularly when children require hospitalisation for complications. Complicated SAM is a multisystem disease with high inpatient and post-discharge mortality, especially in children with co-morbidities such as HIV; however, the underlying pathogenesis of complicated SAM is poorly understood.

Targeted multiplex biomarker analysis in children hospitalised with SAM (N=264) was conducted on plasma samples, and inflammatory markers assessed on stool samples, taken at recruitment, discharge, and 12-24- and 48-weeks after discharge from three hospitals in Zimbabwe and Zambia. Compared with adequately-nourished controls (N=173), we found that at baseline, complicated SAM is characterised by systemic, endothelial, and intestinal inflammation, which is exacerbated by HIV infection. This persists over 48 weeks despite nutritional recovery, and is associated with children’s outcomes. Baseline plasma concentrations of vascular endothelial growth factor, glucagon-like peptide-2, and intestinal fatty acid binding protein are independently associated with lower mortality or hospital readmission over the following 48 weeks. Following principal component analysis of baseline biomarkers, higher scores of a component representing growth factors is associated with greater weight-for-height Z-score recovery and lower mortality or hospital readmission over the 48 weeks. Conversely components representing higher gut and systemic inflammation are associated with higher mortality or hospital readmission.

These findings highlight the interplay between inflammation, which damages tissues, and growth factors, which mediate endothelial and epithelial regeneration. This supports further studies investigating interventions to reduce inflammation and promote epithelial repair as an approach to reduce mortality and improve nutritional recovery.

## Introduction

Undernutrition is a major global health concern, underpinning 45% of deaths among children under five-years-old every year (1). Correspondingly, the United Nations Sustainable Development Goal 2 aims to end malnutrition in all its forms by 2030 (2). Severe acute malnutrition (SAM) is the most life-threatening form of undernutrition and is defined as a weight-for-height z-score less than -3, mid-upper-arm circumference less than 115mm, or the presence of bilateral nutritional edema (3). Where there are no co-existing complications, children with SAM are treated in the community; however, if children have a concomitant infection, suppressed appetite, or severe edema, they require admission to hospital and are defined as having complicated SAM. Mortality in complicated SAM remains high: in a recent meta-analysis of children under five years old who were hospitalised with SAM, the mean inpatient mortality from studies since 2000 was 15.7%, (4) which substantially exceeds the 5% mortality previously stated as ‘good’ by the WHO (5).

Although the term ‘acute’ in severe acute malnutrition reflects the fact that many children present to hospital with short-term complications, this nomenclature overlooks the chronicity of underlying undernutrition and its long-term impact on morbidity and mortality. The Health Outcomes and Pathogenesis of SAM (HOPE-SAM) study, which followed children for 48 weeks after admission for SAM, showed that the outpatient mortality of 9.1% nearly matched the 9.4% inpatient mortality (6), and 16% of children were readmitted to hospital after discharge (7). Deaths are frequently attributed to infections, and children are therefore considered to be functionally immunosuppressed, with increased systemic inflammatory markers (8). Children surviving SAM have been shown to have impaired growth and physical and cognitive function even several years later, particularly in settings with a high human immunodeficiency virus (HIV) burden (9). Recognising this chronicity, the United Nations has begun interchangeably to use the term ‘severe wasting’ (10), and others to use the term ‘severe malnutrition’ (11) to describe SAM. Although the wasting reflects long-term nutrient deprivation, edematous malnutrition is virtually always acute.

Despite the high mortality burden, the underlying pathophysiology of SAM is poorly understood. The disease is systemic and affects multiple organs, immunity, and metabolism. It is known that children with SAM have substantial structural and functional gut abnormalities, termed malnutrition enteropathy, characterised by small intestinal inflammation, abnormal villus architecture, increased gut permeability, and malabsorption (12). This can allow epithelial breaches and microbial translocation from the gut lumen into the systemic circulation (13). It is believed that exposure to antigenic material, including the lipopolysaccharide (LPS) component of the gram-negative bacterial cell wall, induces inflammation and blunts subsequent immune responses to pathogens (14). It is hypothesised therefore that failure of epithelial repair may perpetuate this cycle of inflammation and enteropathy (15). However, few studies have evaluated the impact of these pathogenic pathways on mortality and nutritional recovery, or the timescale of resolution after inpatient management of SAM.

A substantial proportion of children admitted with SAM in sub-Saharan Africa have HIV infection (4), which is associated with four-fold higher inpatient and outpatient mortality compared to children with SAM alone (6). HIV compounds the negative effects of SAM through direct HIV-mediated cellular injury to multiple organs, and indirect effects including chronic immune activation and opportunistic infections arising from the immunosuppressed state (16). These processes reinforce the inflammation, enteropathy and immune dysfunction cycle, making ‘HIV+SAM’ potentially pathophysiologically distinct from SAM alone, and likely contributing to the higher mortality (17).

We conducted a targeted multiplex analysis to characterise biomarkers of inflammation, enteropathy, and tissue repair in children hospitalised for complicated SAM to evaluate: i) whether children with HIV and SAM have more pathological perturbations than children with SAM alone; ii) whether plasma biomarker concentrations in children with SAM normalise to those of the adequately nourished control group over 48 weeks after discharge from hospital; and iii) whether these pathological pathways are associated with mortality, readmission to hospital and nutritional recovery following complicated SAM.

## Results

**Figure 1:**
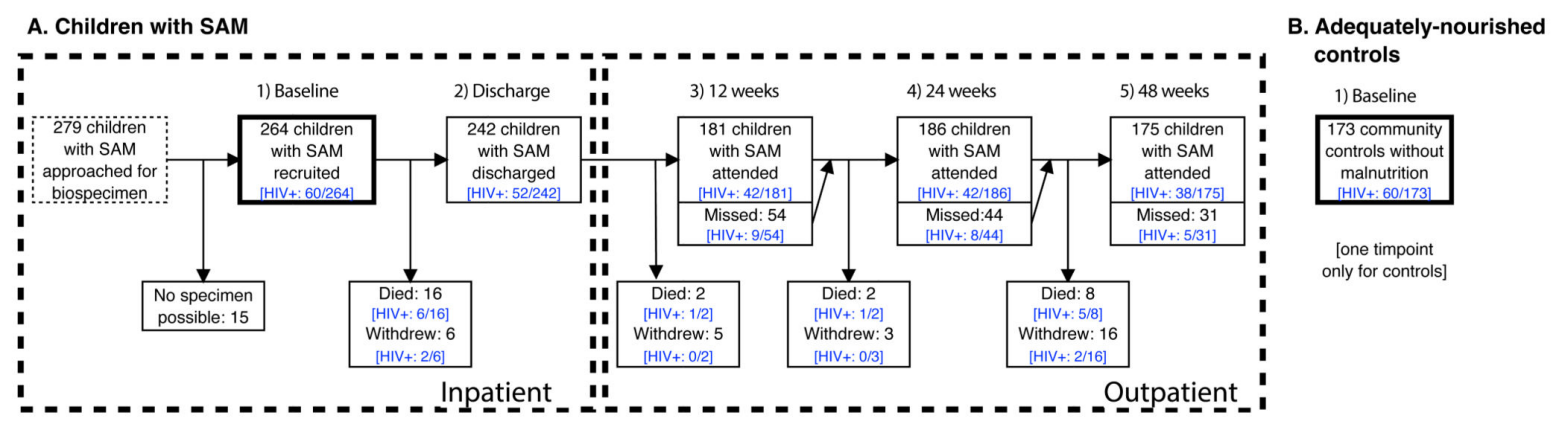
Flow diagram for children recruited to the enteropathy sub-study of the HOPE-SAM longitudinal study (A) 264 children with SAM were recruited in hospital and followed up for 48 weeks after discharge. Participants had stool and blood samples taken at 5 time-points: baseline, discharge, 12 weeks, 24 weeks, and 48 weeks. The number of children who missed each visit are shown as are the numbers of children who died, or permanently withdrew. In addition, there were two visits at 2 weeks and 4 weeks after hospital discharge during which participants had anthropometry only measured. The number of children with HIV in each of the groups is shown in brackets. (B) 173 adequately-nourished controls were recruited and underwent testing at one timepoint only.

We recruited 279 children hospitalised with SAM in Zambia and Zimbabwe to a longitudinal study; 264 (95%) had available biospecimens and were included in the current analysis, of whom 60 (23%) had HIV infection. A cohort of 173 adequately-nourished controls, recruited from the same sites, provided blood, urine and stool samples at a single cross-sectional time-point to compare biomarkers between groups; 60/173 (35%) had HIV infection. Baseline characteristics of both groups are shown in Table 1, with differences between these children and the 15 children we intended to recruit but were unable to provide a biospecimen shown in Table S1. Follow-up over 48 weeks after discharge for children with SAM is shown in Fig. 1. Over the study period, 28 (11%) children died, 30 (11%) children withdrew, and a further 26 (10%) children were re-admitted to hospital after discharge.

### SAM in hospitalised children is characterised by inflammation and enteropathy

**Figure 2:**
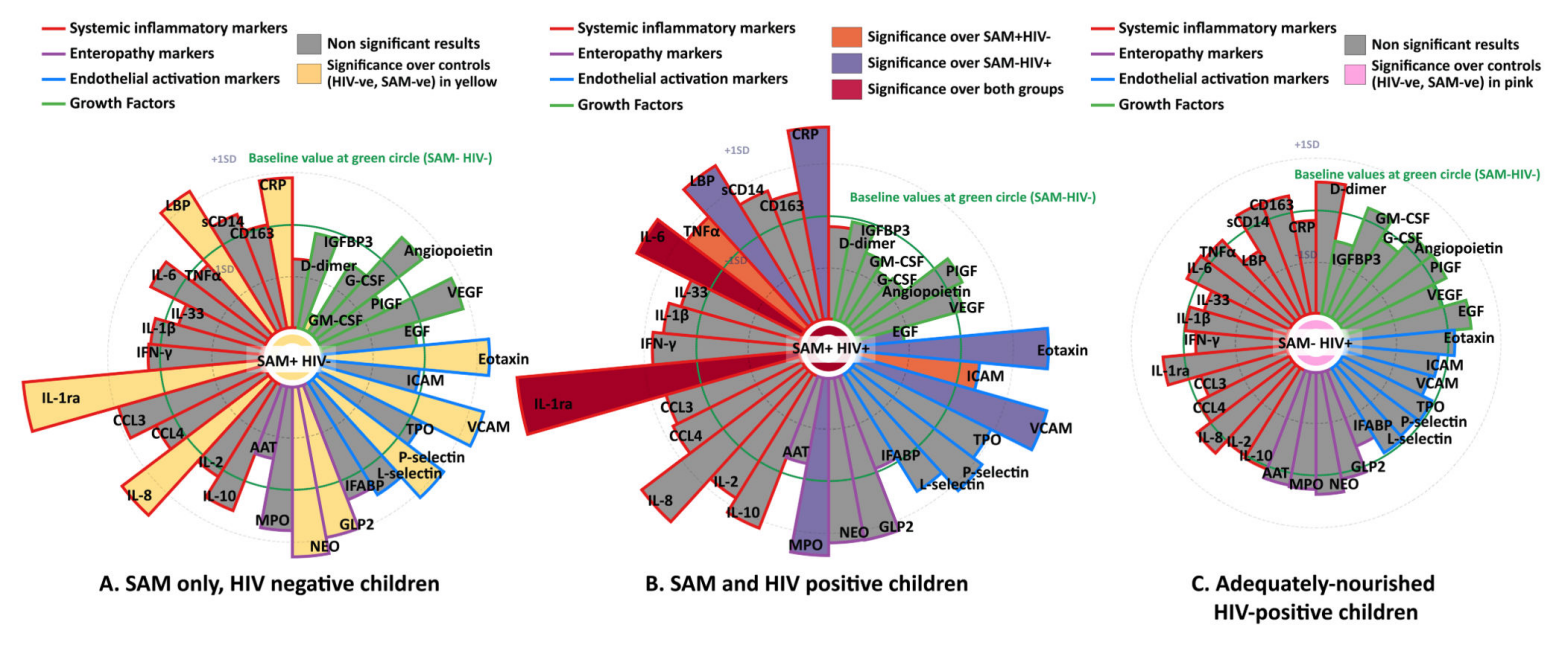
Comparison of baseline biomarkers concentrations between children with SAM and adequately-nourished children, and the differences in seen in children living with on each of these groups. A) The results of biomarkers from children with SAM and without HIV are compared with adequately-nourished HIV-negative children. B) The results of biomarkers from children with SAM and living with HIV C) The results of biomarkers from adequately-nourished children living with HIV compared with adequately-nourished children not living with HIV. The biomarker values of the adequately nourished HIV-negative children (‘baseline value’) are shown at the point of the green circle on each graph. The standard deviation (+1SD, and -1SD) of the baseline value is shown on the graphs. Each ray represents one biomarker (labelled). If the ray is larger than the green circle, that biomarker is increased in that group compared to the baseline value. If it is smaller, it is lower in that group compared to the baseline value. If it is shaded, the difference is statistically significant, as per corrected calculation below. All biomarkers were log_10_ transformed prior to analysis. Results explore changes in standard deviation from the baseline, meaning the magnitude of changes do not necessarily correlate to absolute pg/mL changes between biomarkers. Comparisons between groups used ordinary least squares regression if biomarker values differed by 1/3 of the standard deviation of the baseline group (adequately-nourished HIV-negative children if present, or the largest group if not). The P-value was adjusted using the Romano-Wolf multiple hypothesis correction method, and was coloured in the graph if the adjusted P<0.05.

We first compared 34 biomarkers of inflammation, enteropathy, endothelial activation and growth, listed in Table S2, between adequately nourished controls and children hospitalised with SAM, stratified by HIV status. Baseline correlations between biomarkers are shown in Fig. S1, and sample missingness profile is shown in Table S3. Biomarkers were first explored to examine the magnitude of difference between case and control groups. Biomarkers with large group differences were further tested to investigate if these differences were significantly different using linear regression, with correction for multiple testing. Children with SAM, compared to adequately nourished controls, had higher systemic inflammatory markers, including C-reactive protein (CRP), LPS-binding protein (LBP), interleukin (IL) 8 (IL-8), and interleukin-1 receptor antagonist (IL-1ra), which is typically higher in inflammation despite its anti-inflammatory function), endothelial activation markers [eotaxin, vascular cell adhesion molecule 1 (VCAM-1), and P-selectin] and reduced granulocyte-macrophage colony-stimulating factor (GM-CSF) in their plasma samples (Fig. 2A). Among enteropathy markers, children with SAM had higher plasma glucagon-like peptide-2 (GLP2) and fecal neopterin compared to adequately nourished children. Findings were broadly similar across HIV strata (Fig. 2; Table S4).

### HIV exacerbates inflammation and endothelial activation in children with SAM

Given the poorer outcomes and higher frequency of complications among children with HIV and SAM together (HIV+SAM) compared with SAM alone, we next explored differences in biomarkers between these groups during hospitalisation, after correcting for multiple hypothesis testing. Four biomarkers were differentially expressed in children with HIV+SAM compared to children with SAM (Fig. 2B, shaded orange and red). Children with HIV+SAM, compared to SAM alone, had higher concentrations of tumour necrosis factor alpha (TNFα), IL-6, and IL-1ra. There was also evidence of more endothelial activation, as indicated by higher concentrations of intercellular adhesion molecule-1 (ICAM-1). By contrast, there was no significant difference in any biomarker between adequately nourished controls when compared by HIV status (Fig. 2C).

### Systemic, vascular, and intestinal inflammation do not resolve over 48 weeks

**Figure 3:**
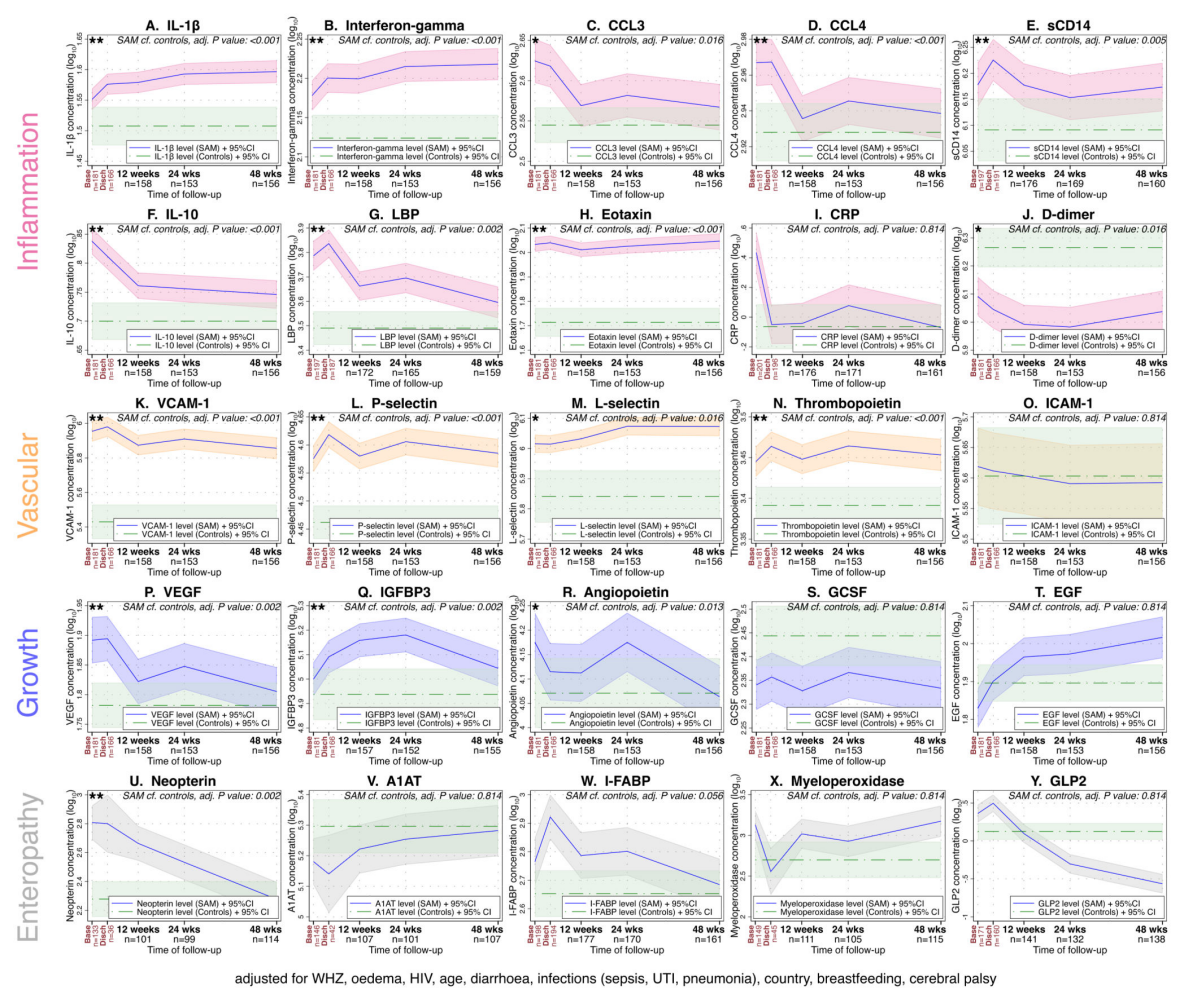
Longitudinal results of biomarkers of inflammation, endothelial activation, growth, and enteropathy from baseline to 48 weeks Biomarkers measured during inpatient stay at baseline and discharge (shown in red text on the graph), and at 12 weeks, 24 weeks, and 48 weeks after discharge. The number of samples analysed at each timepoint is shown. Biomarker concentrations are shown for systemic inflammation (pink): A) IL-1β, B) interferon-gamma, C) chemokine ligand 3 (CCL3), D) CCL4, E) soluble CD14, F) IL-10, G) lipopolysaccharide binding protein (LBP), H) eotaxin, I) C-reactive protein (CRP), and J) D-dimer. Vascular activation marker concentrations (gold) shown include K) vascular cell adhesion molecule 1 (VCAM-1), L) P-selectin, M) L-selectin, N) thrombopoietin and O) intercellular adhesion molecule-1 (ICAM-1).. Growth factor concentrations (blue): P) vascular endothelial growth factor (VEGF), Q) insulin-like growth factor binding protein 3 (IGFBP3), R) angiopoietin, S) granulocyte-colony stimulating factor (GCSF), and T) epidermal growth factor (EGF). Enteropathy marker concentrations (grey): U) neopterin, V) alpha-1-antitrypsin (A1AT), W) intestinal fatty-acid binding protein (I-FABP), X) myeloperoxidase, and Y) glucagon-like peptide 2 (GLP2). The baseline non-SAM healthy control concentration, measured once, is shown by the dashed green line on each graph. The results were analysed using mixed effects modelling, with the log_10_ biomarker, HIV status, baseline oedema, WHZ score, age, presence of diarrhoea at admission [yes/no], infections (sepsis, UTI, pneumonia), country, breastfeeding, and cerebral palsy being offered to the model as fixed effects, and participant identifier as the random effect. The number of results at each time point for each biomarker is shown for children with SAM. The P-value displayed is the value from this model for the difference between the longitudinal value for cases, and the baseline healthy value, adjusted for multiple hypothesis testing using the Romano Wolf adjustment. ** P < 0.01; * P < 0.05; 95% CI – 95% confidence interval. Full results are shown in Table 2.

The longitudinal changes in individual biomarkers among children with SAM from inpatient enrolment through 48 weeks post-discharge is shown in Fig. 3. These results were following correction for multiple hypothesis testing, and adjustment for the 12 covariates stated in Fig. 3, which were chosen by selecting the minimum sufficient adjustment set from a directed acyclic graph (Fig. S2) full results are shown in Table 2. Over the study period, children with SAM had higher plasma concentrations compared to adequately nourished controls of 14 out of the 17 systemic inflammatory biomarkers including IL-1β [Fig. 3A], interferon-gamma (IFN-γ) [Fig 3B], chemokine ligand 3 (CCL3) [Fig 3C], chemokine ligand 4 (CCL4) [Fig 3D]; soluble CD14 [Fig 3E], IL-10 [Fig 3F], LBP [Fig 3G], and eotaxin [Fig 3H]. CRP, although higher at baseline, was not significantly higher over the study period [Fig 3I]. Only D-dimer was significantly reduced in children with SAM compared with controls over the whole period (p=0.016) [Fig 3J]. Four out of five vascular inflammatory markers were also significantly raised including VCAM-1 [Fig 3K], P-selectin [Fig 3L], L-selectin [Fig 3M], and thrombopoietin [Fig 3N], but not ICAM-1 [Fig 3O].

The majority of circulating growth factors were not significantly different between children with SAM and controls over 48 weeks. However, vascular endothelial growth factor (VEGF) [Fig 3P], insulin-like growth factor binding protein 3 (IGFBP3) [Fig 3Q], and angiopoietin [Fig 3R] were higher in children with SAM, suggesting an ongoing attempt at epithelial restoration as well as potential vascular angiogenesis/regeneration. GCSF [Fig 3S] and EGF [Fig 3T] were not significantly different over the 48 weeks.

Of the enteropathy markers, only neopterin was significantly different, being higher in cases than among controls (p=0.002) [Fig 3U]; however, many other enteropathy markers changed over time in children with SAM. Three out of the six enteropathy markers (alpha-1-antitrypsin (A1AT) [Fig 3V], neopterin [Fig 3U], and intestinal fatty acid binding protein (I-FABP) [Fig 3W]) normalised to the concentration of adequately nourished controls by 48 weeks post-discharge, with myeloperoxidase [Fig 3X] and GLP2 [Fig 3Y] remaining altered.

A sensitivity analysis to explore the missing data assumption implemented did not affect the inferences (Table S5), further strengthened by there being no difference in baseline demographic data between children with a sample at week 48 and those without (Table S6). Taken together, these data highlight the persistence of vascular, systemic and intestinal inflammation in children recovering from SAM, up to 48 weeks after their initial hospitalisation.

### Principal component analysis identifies several distinct pathogenic processes in SAM

**Figure 4:**
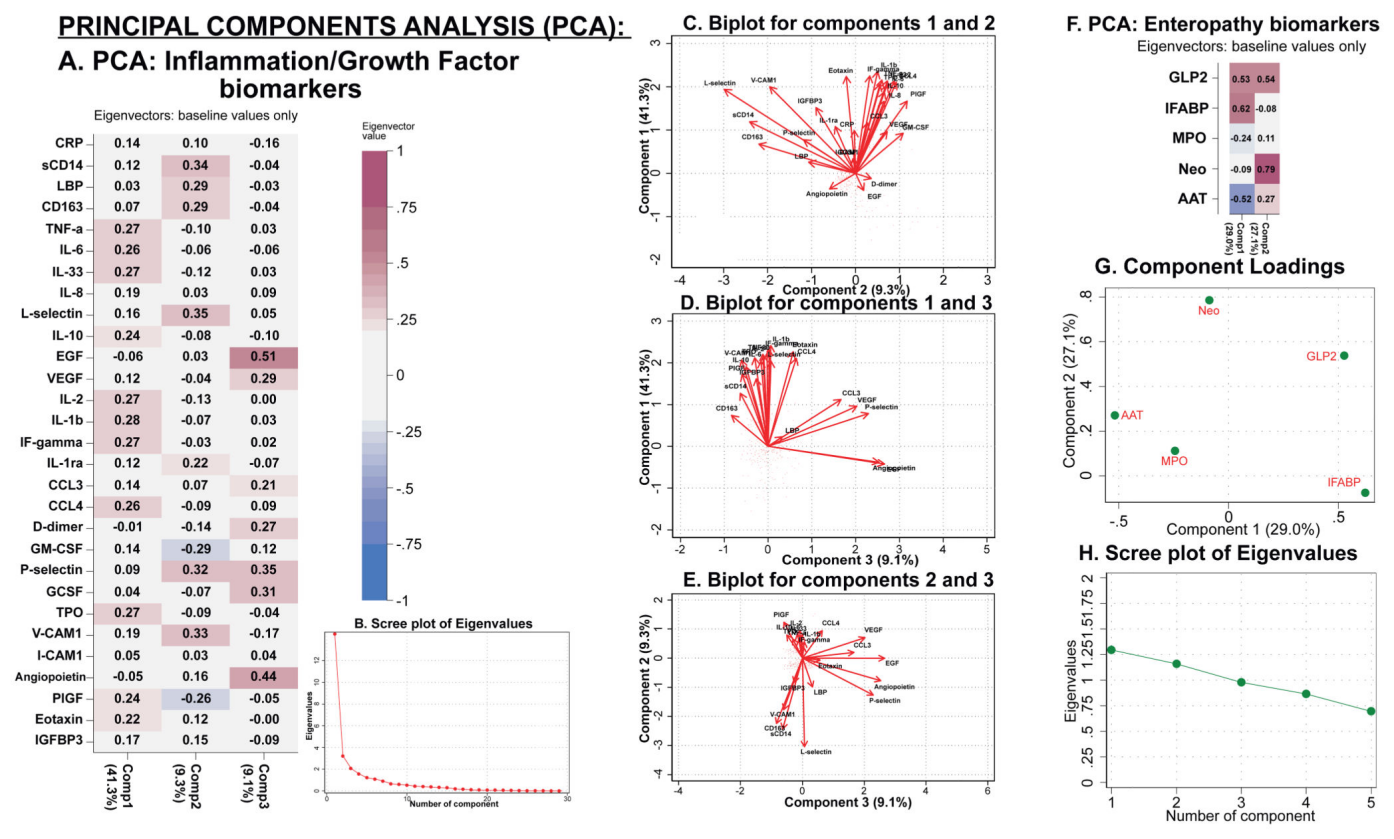
Principal component analysis of baseline biomarkers Component loadings for the inflammatory/growth biomarkers are shown in A) to produce ‘Systemic Components’, along with B) the eigenvalues in the scree plot. The biplots of C) Systemic Components 1 and 2, D) Systemic Components 1 and 3, and E) Systemic Components 2 and 3 are shown to show the different effects of the individual biomarkers. Principal component analysis was undertaken separately for the enteropathy markers in F) to produce ‘Gut Components’, along with G) the component loadings and H) the eigenvalues for the Gut Components.

To reduce data dimensionality among the inflammatory, endothelial, and growth factor markers, a principal component analysis (PCA) identified three ‘systemic’ biomarker components(Fig. 4, A to E). The first component (Systemic Component 1) comprised systemic inflammatory markers, largely involved in host responses to pathogens, including IL-1β, IL-6, TNFα, IL-10, and IFN-γ; additional inflammatory markers included eotaxin, chemokine ligand 4 (CCL4), and IL-33 (Fig 4A; 4C-D). The second component (Systemic Component 2) represented inflammation and endothelial activation and included L-selectin and P-selectin, and markers of monocyte/macrophage activation in response to LPS exposure, including soluble CD14 (sCD14), CD163, and LBP (Fig 4A; 4C; 4E). The third component (Systemic Component 3) comprised positive contributions from growth factors [EGF, VEGF, D-dimer, granulocyte-colony stimulating factor (GCSF), and angiopoietin] and smaller negative contributions from the systemic inflammatory marker CRP, inflammatory endothelial markers, and VCAM-1 (Fig 4A, 4D-E).

We conducted a separate PCA of the enteropathy biomarkers to investigate the role of the gut in driving the systemic biomarker differences observed. We identified two ‘gut’ components (Fig. 4, F to H). The first (Gut Component 1) component included strong positive contributions from plasma GLP2 and intestinal fatty acid binding protein (I-FABP), and negative contributions from faecal myeloperoxidase (MPO) and fecal A1AT (Fig 4F). This component putatively represents epithelial repair in response to small intestinal villus damage, because I-FABP is released into the circulation following damage to enterocytes, and GLP2 is an intestinotrophic hormone promoting epithelial proliferation/regeneration (18, 19). The second component (Gut Component 2) included strong positive contributions from A1AT, neopterin and GLP2, suggesting intestinal inflammation and potential loss of gut barrier function in children with SAM, triggering GLP2-mediated regeneration (Fig 4F). Components did not significantly change over time (Fig. S3).

### Low baseline growth factors are associated with reduced nutritional recovery over 48 weeks

**Figure 5:**
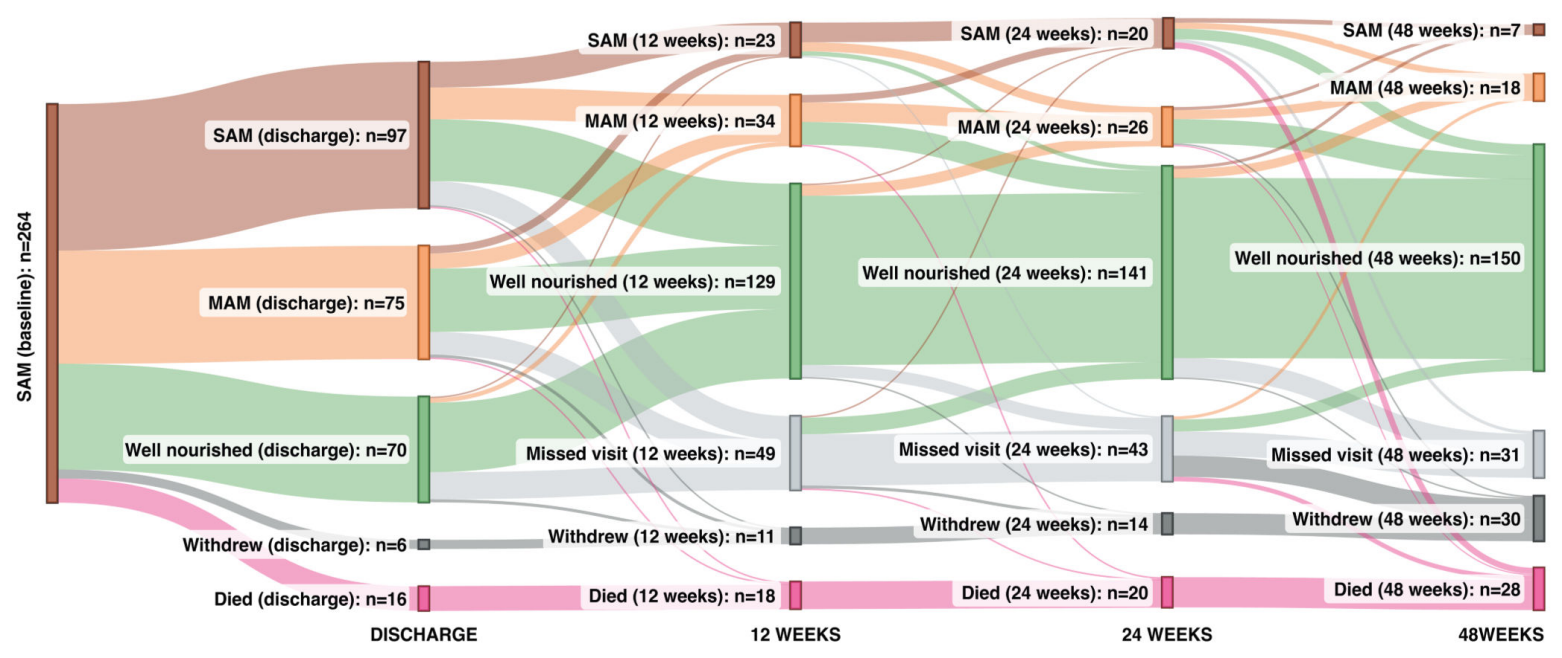
Sankey diagram showing longitudinal nutritional status of children enrolled with SAM over 48 weeks after discharcge All children were enrolled during hospitalisation with complicated SAM, and following inpatient management, children were discharged with SAM (weight-for-height z-score (WHZ) < -3 or bilateral pitting edema), moderate acute malnutrition (MAM; WHZ < -2), or well-nourished (WHZ > -2), according to World Health Organization definitions. Overall, 78/264 (29.8%) children had failed nutritional recovery, defined as a worsening of their nutritional category from one timepoint to a subsequent one, or death.

The nutritional and vital status of children at each timepoint is shown in the Sankey diagram in Fig. 5, with average levels of WHZ at each timepoint shown in Fig. S4. To identify which patterns of biomarkers during hospitalisation were associated with subsequent growth recovery, we first evaluated whether baseline values of each principal component were associated with change in weight-for-height z-score (WHZ) over 48 weeks, a measure previously associated with mortality in children with SAM (4). In a mixed effects model, each unit increase in Systemic Component 3, which largely comprised growth factors, was associated with a 0.24 (95% confidence interval (95%CI) 0.10, 0.37) increase in WHZ over the following year. By contrast, other Systemic and Gut Components were not significantly associated with changes in WHZ over 48 weeks. Taken together, this suggests that a range of baseline growth factors are associated with greater tissue accretion over 48 weeks following complicated SAM.

We next explored categorical changes in nutritional status over time, using categories of SAM, moderate acute malnutrition (MAM) and adequately nourished to define children at each time-point. Children who had failed to recover nutritionally at any point (defined as a having worse nutritional category than at a previous timepoint, or dying) had a lower concentration of the growth factor binding protein IGFBP3 (-0.22 log_10_ pg/mL, 95%CI -0.33, - 0.10, p=0.010) following Romano-Wolf correction for multiple hypothesis testing (Table S7). Although this analysis should be interpreted with caution, because it required a participant to have a subsequent timepoint to be included in the analysis, it suggests a role for this protein which prolongs the half-life of the insulin-like growth factors and inhibits the positive effect of humanin on insulin sensitivity (20).

### Systemic inflammation, vascular inflammation, and a lack of epithelial regeneration are associated with mortality and hospital readmission

**Figure 6:**
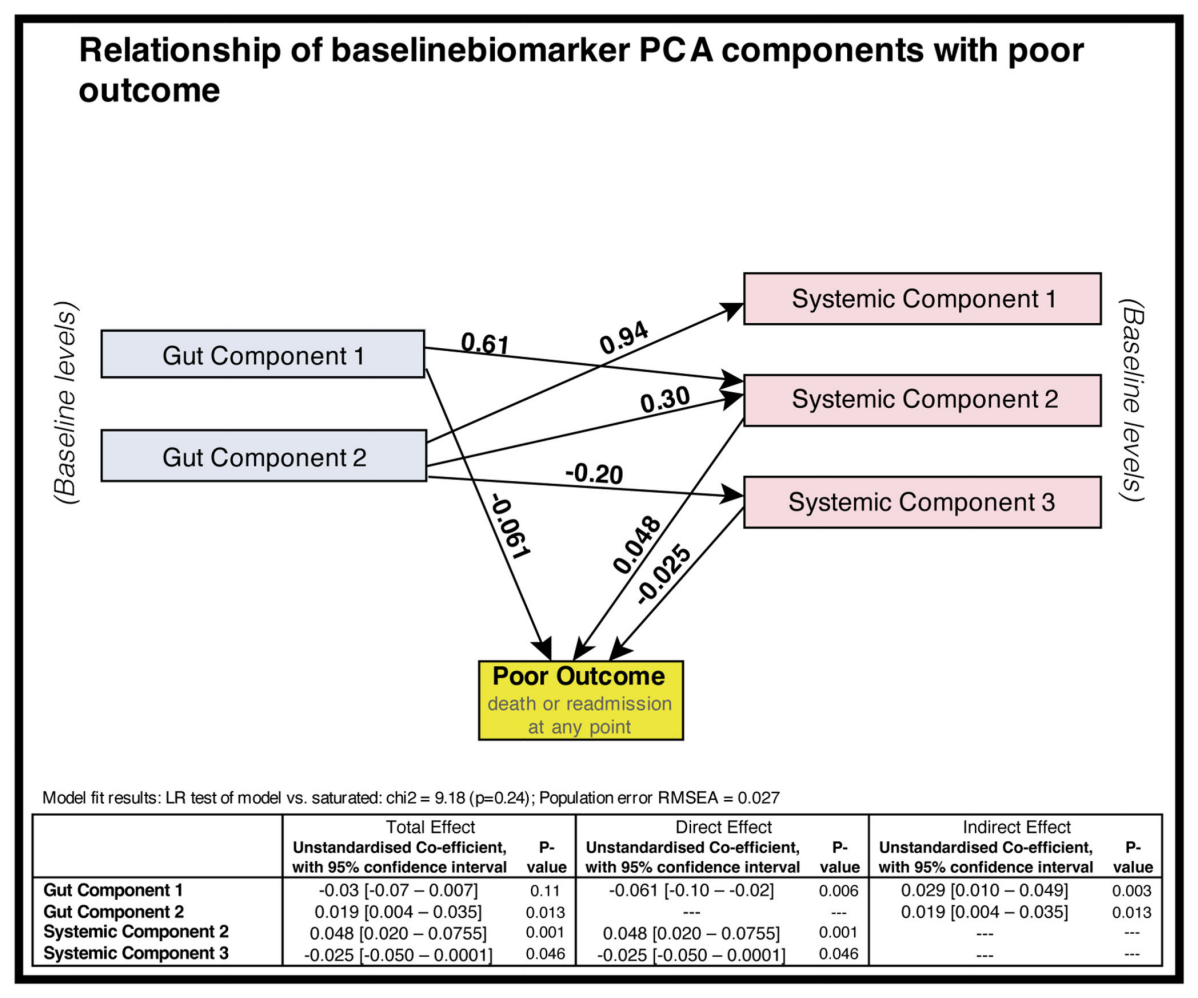
Structural equation models (SEM) examining the relationship of the baseline Gut Components with the Systemic inflammatory components SEM modelling was carried out to examine the structural relationship between baseline biomarkers following PCA analysis (as described in Fig. 4) and a poor outcome. The model tested was the base model shown in Fig. S6. An arrow shows a relationship between those variables with statistical significance defined as P < 0.05. Gut Components had both a direct effect and an indirect effect via their effect on Systemic Components with a poor outcome, defined as death or readmission at any point. Higher scores for Gut Component 2, largely composed of biomarkers reflective of gut inflammation, have a negative association with the levels of Systemic Component 3, largely composed of growth factors. Higher scores for this Systemic Component 3 were associated with better outcomes, whereas higher scores for Systemic Component 2, largely composing of pro-inflammatory markers, were associated with poorer outcomes. Missing data were handled by the SEM modelling using using full imputation maximum likelihood; all children were therefore included in the model (n=264). The total effects of any of the variables with a poor outcome are included in the table, split into direct and indirect effects. Non-standardized coefficients are displayed.

Given the high rates of mortality and readmission to hospital previously reported in the HOPE-SAM cohort (6, 7), we next investigated whether biomarkers measured during hospitalisation predicted poor clinical outcomes over the following year. Fig. 6 shows the results of a structural equation model (SEM) examining the relationship between each baseline principal component (three ‘systemic’ components, and two ‘gut’ components) and a composite outcome of death or readmission to hospital (n=56). In unadjusted models, a higher Gut Component 2 and higher Systemic Component 2 were both independently associated with death or readmission. Because both of these components represent systemic inflammation or inflammation/damage in the gut, this suggested a deleterious effect of chronic inflammation during recovery from SAM. Conversely, Systemic Component 3 (which largely comprises growth and tissue repair factors), was independently weakly associated with a lower risk of death or readmission (Fig. 6). There was also some evidence that greater epithelial repair, largely represented by Gut Component 1, was associated with better outcomes: on estimating direct effects in the SEM model, there was a significant reduction in death or readmission (p=0.006). There were also several independent relationships between components: markers of gut inflammation/damage (Gut Component 2) were associated with higher systemic inflammation (Systemic Components 1 and 2), and lower vascular/endothelial growth factors (Systemic Component 3) (Fig. 6).

### VEGF, GLP2, and I-FABP are independently associated with reduced mortality and hospital readmission

We next explored whether any individual biomarker was associated with mortality or hospital readmission. Each of the biomarkers contributing factor loadings of >0.20 in the Systemic Components and identified as clinical predictors were included in a Cox regression model (full results shown in Table 3). In univariable analysis, higher concentrations of the growth factors EGF [Hazard ratio (HR) 0.61, 95%CI 0.39, 0.95], VEGF [HR 0.45, 95%CI 0.26, 0.76], and angiopoietin [HR 0.73, 95%CI 0.54, 0.98] were associated with a lower risk of death or readmission. In multivariable analysis, accounting for variables selected using the minimum sufficient adjustment set from a directed acyclic graph (Fig. S5), each log rise in VEGF was associated with a 50% reduction in mortality or readmission [aHR 0.50, 95%CI 0.28, 0.89], whereas other systemic biomarkers were not independently predictive. This suggests that VEGF, which has pleiotropic roles in epithelial repair in addition to its well-recognised angiogenic properties (21), may be promoting tissue repair during recovery from SAM.

Gut Component 1, which was associated with mortality or readmission, contains four individual biomarkers contributing component loadings of over 0.20. A univariable Cox regression model including these biomarkers showed that higher concentrations of GLP2 [HR 0.81, 95%CI 0.67, 0.99] and I-FABP [HR 0.80, 95%CI 0.65, 0.99] were associated with lower mortality or readmission. After adjusting for confounders, GLP2 and I-FABP remained independently predictive of clinical outcomes: each log rise in GLP2 was associated with a 25% reduction in mortality or readmission [aHR 0.75, 95%CI 0.61, 0.93] and each log rise in I-FABP was associated with a 23% reduction in mortality or readmission [aHR 0.77, 95%CI 0.62, 0.97].

Taken together, our findings highlight the inter-related processes that are apparent in children with SAM, with evidence of persistent inflammation which can drive tissue damage, and concurrent attempts to repair epithelial surfaces. The outcome of this interplay, which is disrupted by HIV infection, may determine the ability to restore critical barrier function at mucosal surfaces, ultimately enable tissue accretion, and is associated with the risk of death or readmission to hospital.

## Discussion

This longitudinal study of children hospitalised with complicated SAM in Zambia and Zimbabwe has three major findings. First, we showed that SAM is characterised by inflammation in multiple compartments that does not resolve over 48 weeks after discharge, despite phenotypic recovery. Second, we showed that a suite of growth factors involved in angiogenesis, cell proliferation and tissue repair, including VEGF and EGF, are detectable during hospitalisation for SAM, and influence WHZ recovery and mortality over the following year. Third, we showed that biomarkers indicating higher systemic inflammation and lower epithelial regeneration are associated with a greater risk of death and readmission to hospital. The pattern of biomarkers during hospitalisation for SAM identified by PCA therefore highlights the competing processes of inflammation and tissue regeneration, which may influence mortality, readmission and growth recovery during convalescence.

Chronic inflammatory processes, apparent in the gut, vasculature and systemic circulation, are typically associated with drive tissue remodelling and loss of normal epithelial function (22); by contrast, growth factors can promote epithelial repair, restoration of barrier function and tissue accretion. This interplay suggests multiple potential targets for therapeutic approaches in this high-risk condition. Interventions targeting inflammatory processes, augmenting epithelial repair, or both, could plausibly shift the balance of tissue damage and restoration, and improve long-term clinical outcomes.

Inflammatory markers are elevated during hospitalisation for SAM and may partly reflect the child’s response to infections. Current treatment protocols include universal antibiotics: children with uncomplicated SAM treated in the community have reduced mortality if they receive broad-spectrum oral antibiotics (23), and those with complicated SAM receive parenteral antibiotics because infections such as sepsis, pneumonia and diarrhea are common and are associated with mortality (24). Infections may therefore underlie increased baseline concentration of CRP which declined in the SAM cases to concentrations similar to the adequately nourished control group after their discharge from hospital. However, infections may not explain the full spectrum and the persistence of elevated inflammatory markers in SAM, including IL-1β, IFN-γ, sCD14, and LBP, as well as markers of endothelial activation such as VCAM-1, P-selectin, L-selectin and thrombopoietin. The complex pattern of inflammation represented by these markers is likely to be multifactorial. Chronic exposure to immunogenic stimuli can lead to a refractory period of immune dysfunction, or immunoparalysis in other contexts, such as multiple organ dysfunction syndrome (25). Elevated inflammatory markers persisted in the SAM group over at least 48 weeks following hospital discharge. A chronic state of inflammatory immunoparalysis may therefore contribute to the high post-discharge mortality in children with complicated SAM, despite the recovery of WHZ. Longer term, the persistence of systemic and vascular inflammation may underlie the long-term non-communicable disease risk seen among survivors of SAM (9).

SAM is characterised by profound enteropathy (26), with infiltration of lymphocytes into the lamina propria (27), leading to loss of mucosal barrier function and microbial translocation, which appears to drive systemic and vascular inflammation. A reinforcing cycle may also occur, because the systemic inflammatory response has been shown to increase intestinal permeability in animal models (28). It is notable that even adequately nourished controls have a substantial degree of enteropathy, because the biomarkers contributing to Gut Component 1 from the principal component analysis did not differ significantly between cases and controls. This likely reflects environmental enteropathy, an alteration in gut structure and function that overlaps and interacts with other causes of enteropathy in this setting (12). Based on the SEM modelling, enteropathy appears to drive pathology and poor clinical outcomes once children develop SAM, which may reflect an inability to compensate for loss of gut barrier function once children develop severe tissue depletion and multi-system dysfunction.

Systemic plasma concentrations of growth factors, including EGF and VEGF, increased over 48 weeks of follow-up in this cohort. This likely reflects epithelial repair, an increase in muscle mass and the vascularisation that is required for both processes. EGF is secreted from submaxillary glands, activated platelets, and from Brunner glands of the duodenum, and is the most abundant growth factor in breast milk. It is involved in promoting cell proliferation and has effects on the gut, inducing intestinal repair, enhancing enterocyte proliferation, and stimulating secretion of digestive enzymes (29). VEGF is a platelet-derived angiogenic growth factor that is secreted to increase haematopoiesis, wound healing, bone growth, or muscle growth, encouraging new blood vessels to form. This drive to proliferation and growth is likely to explain why baseline VEGF and EGF concentrations are associated with increased WHZ scores during convalescence.

The balance between inflammation and tissue regeneration is highlighted in the SEM, which showed that pro-inflammatory systemic markers are associated with increased mortality or readmission, whereas growth factors are associated with lower mortality or readmission. The suite of growth factors detected in children with SAM could reflect an attempt to restore normal barrier function or increase tissue growth (21). The extent of epithelial damage in SAM is marked (13), and can be directly visualised in the gut of adults with environmental enteropathy as plumes of effluxing fluorescein using confocal laser endomicroscopy (30, 31). We posit that an inadequate growth factor response in the face of intestinal damage and persistent inflammation reflects a lack of epithelial repair, which permits further inflammatory damage and tissue remodelling, and ultimately drives mortality or poor growth recovery. A study from Malawi of 79 children admitted with SAM examined the relationship between 29 admission cytokines and mortality and showed that seven cytokines (GCSF, IL-1ra, IL-6, IL-2, TNFα, TNFβ, and IL-13) were higher in children who died compared to those who recovered, and that both systemic and intestinal inflammation (modulated by short-chain fatty acids) are associated with mortality (32). A Kenyan study showed that inflammatory proteins such as CRP, LBP, TNFα, IL-8, IL-15, IP10, and GCSF are increased in children with SAM who died within 60 days of discharge from hospital compared with those who survived to 48 weeks (33). In addition to systemic inflammation, von Willebrand factor was independently associated with mortality, supporting the importance of endothelial activation. Interventions to reduce inflammation and promote epithelial restoration in tandem may plausibly reduce mortality and improve recovery. A Kenyan study trialled mesalazine in children with SAM (34), and showed some promise in reducing systemic inflammation by targeting the gut. In Zimbabwe and Zambia, an ongoing phase II intervention trial among children hospitalised with SAM (35) is evaluating the GLP2 analogue teduglutide to promote intestinal epithelial regeneration, and the non-absorbed steroid budesonide to reduce intestinal inflammation.

The main strength of this study is the longitudinal collection of blood and stool from a large cohort of children hospitalised with SAM across three centres in southern Africa, and the use of multiplex panels to evaluate systemic, vascular and intestinal pathology compared with adequately nourished controls. Limitations include incomplete biomarker data (as summarised in Table S8), either due to sampling difficulty or missed visits, although our sensitivity analysis suggested the missing data had minimal impact on our interpretation, demographics of children with and without a sample at week 48 were not significantly different, and the full-imputation maximum likelihood for SEM yielded similar inferences. In addition, children were generally recruited 24-48 hours after admission to hospital, given the time taken by caregivers to consider study recruitment, meaning our cohort was not fully representative of complicated SAM, since the sickest children may have died before enrolment, as highlighted by the baseline differences between children with and without available biological specimens. Our control cohort, comprising adequately nourished children without acute infections, was designed to provide normative biomarker data, but enrolment from hospital clinics may have introduced bias compared to sampling of healthy community controls.

In conclusion we showed children hospitalized with SAM have elevated inflammatory markers that appear to be driven by enteropathy, and this persisted over 48 weeks post-discharge, with HIV exacerbating intestinal and systemic inflammation in children with SAM. The interplay between inflammation and epithelial repair appears to shape clinical outcomes. Characterising the role of inflammation in SAM pathogenesis, and tissue growth and repair responses in the face of this, is critical to finding new targets for therapeutic interventions to improve outcomes. Results from the current study suggest that longer-term strategies will be needed to target persistent dysregulation of systemic and intestinal processes for at least 48 weeks post-discharge, to reduce the ongoing high mortality in children recovering from complicated SAM.

## Materials and Methods

### Study Design and Participants

This was a sub-study within the Health Outcomes, Pathogenesis and Epidemiology of Severe Acute Malnutrition (HOPE-SAM) study, which has been described in detail elsewhere (36). The HOPE-SAM protocol, standard operating procedures, and case report forms are available at https://osf.io/29uaw/. Briefly, HOPE-SAM was a longitudinal observational cohort study of children hospitalised for SAM at three tertiary referral hospitals in Zambia and Zimbabwe between August 2016 and March 2018. Eligible children were younger than 60 months of age and admitted to a medical ward with complicated SAM, defined as WHZ < -3 or mid-upper-arm circumference (MUAC) <115mm and/or the presence of bilateral nutritional edema (in children above six months of age), according to WHO criteria. Children with malignancy, who died before study enrolment, or whose caregiver was not willing to learn the child’s HIV status were not included in the study. This sub-study enrolled children aged 6-59 months, stratified by HIV status, to more intensive longitudinal specimen collection during follow-up; because the goal was to evaluate biomarkers of enteropathy, children with chronic gastrointestinal diseases were excluded.

Baseline demographic and clinical information was collected on case report forms. Baseline samples were collected following recruitment to the study, and not on the day of admission to hospital, due to the time taken for caregivers to consider informed consent and study enrolment. Inpatient care was provided using WHO country-adapted guidelines for management of complicated SAM. Children were seen in a dedicated study clinic at 2, 4, 12, 24, and 48 weeks after hospital discharge. At each visit, children underwent a clinical examination and anthropometric measurement, including weight, height, MUAC, and head circumference, and history of illness and hospital readmission was obtained. Stool and plasma samples were collected from sub-study children at baseline, discharge, 12, 24, and 48-week visits. Children also had urine collected before and after administration of a solution of lactulose and mannitol, at baseline, discharge, 12 and 48 weeks, as previously described (36).

To provide normative biomarker data for the local population, single samples of blood, stool and urine (pre- and post lactulose-mannitol administration) were collected from adequately nourished control children receiving inpatient or outpatient care at each site. Controls were aged 6-59 months, with WHZ>-1, no acute illness or current infections, and known HIV status. Controls were matched within age bands (6-11 months, 12-23 months, 24-59 months) and by HIV status to enrolled sub-study children with SAM.

### Ethics

Ethical approval for the study was obtained from the University of Zambia Biomedical Research Ethics Committee (010-02-16), and the Medical Research Council of Zimbabwe (MRCZ/A/2044). The ethics committee of Queen Mary University of London provided an advisory review. Written informed consent from caregivers was obtained in local languages.

### Sample size

Our sample size was based on a comparison of 100 HIV-negative children with SAM versus 100 HIV-negative controls, and 100 HIV-positive children with SAM versus 100 HIV-positive controls. Comparing 100 vs. 100 children with a two-sided alpha of 0.025 provided >80% power to detect differences in mean log_10_ inflammatory markers of at least 0.44 standard deviations between groups. For children with SAM, we estimated a composite outcome of mortality at any time in the study (20%) or readmission to hospital following discharge (20%). With an estimated composite adverse outcome in 40% of participants, 200 children with SAM provided 80% power at 5% significance to detect an absolute difference in poor outcomes of 20% (a risk ratio of 2, or odds ratio of 2.67) between those with higher versus lower biomarkers, assuming an elevated biomarker concentration in 50% of participants.

### Laboratory analyses

#### Plasma ELISAs

Plasma was analysed using ELISA according to the manufacturer’s specification for the following analytes: CRP and sCD14 following 1:200 dilution, and sCD163 following 1:40 dilution (R&D Quantikine); LBP following 1:800 dilution (R&D Duoset); I-FABP following 1:5 dilution (Hycult Biotech); and GLP2 at 1:1 dilution (Merck Millipore).

#### Stool ELISAs

A1AT (ImmuChrom) and myeloperoxidase (Immundiagnostik AG) were analysed in stool. 100mg of stool was added to 5mL of wash buffer, and vortexed for 10 minutes, followed by centrifugation for 10 minutes at 3000rpm. The supernatant for A1AT was further diluted 1:250, and myeloperoxidase was further diluted 1:10, and run according to manufacturer’s specifications. Neopterin (Arigobio) was analysed by adding 100mg stool to 0.5mL saline, followed by vortexing for 30mins, and centrifugation. This was then further diluted 1:10 and used in the manufacturer’s assay.

#### Luminex assay

Plasma was analysed in technical duplicate using a custom 25-plex Luminex assay (R&D systems), and run on a Luminex Magpix machine (Luminex Corp). The full range of analytes are shown in Table S2. Results above the limit of detection were repeated at a lower concentration, and results below the limit of detection at the highest concentration (1:2) were included in analyses using a value calculated from the lowest detected result for that analyte divided by √2.

### Statistical analysis

#### Baseline analysis

Differences between groups at baseline (presence or absence of SAM; children with or without HIV) was conducted in a two-step approach to reduce the inflation of type I errors from multiple testing. Firstly, the magnitude of differences of each normalised log_10_-transformed biomarker were assessed between each group, with changes expressed as number of standard deviations away from the baseline group, which was the HIV-negative adequately-nourished group if present, or the largest group if not present. Secondly, to reduce the number of statistical tests, biomarkers were then considered for statistical testing only in those biomarkers with large differences between groups. Because most biomarkers were exploratory, without specific clinical cut-offs, a large difference was defined as more than one-third of a standard deviation away from the baseline group. Statistical hypothesis testing was implemented through ordinary least squares (OLS) regression. The reported P-value was adjusted using the Romano-Wolf multiple hypothesis correction (37). Any difference was considered significant if the adjusted P-value was less than 0.05.

#### Principal component analysis (PCA)

Dimensional reduction of the biomarker data was undertaken using PCA of the systemic inflammatory, endothelial inflammatory/activation, and growth factor biomarkers, and separately for the enteropathy biomarkers. Biomarkers were log_10_ transformed, and then standardized/zero-centred to ensure that the concentration of one biomarker did not have a disproportionate effect on component loadings. Separate PCAs were carried out at each follow-up timepoint to explore whether patterns of biomarkers varied as the child recovered.

#### Survival analysis

Structural equation modelling (SEM) was used to investigate the relationship between the baseline gut and systemic principal components, and a composite outcome of death or readmission to hospital. The analysis was run with and without additional covariates which were selected using a theory-driven approach, following a directed acyclic graph (DAG) that included potential confounders previously shown to be associated with mortality (4), as shown in Fig. S5. The base model was set up as shown in Fig. S6. A full imputation maximum likelihood method was used in the estimation to handle missing data, and significance was defined as P<0.05. The relationship between individual biomarkers and time to mortality or readmission was undertaken using multivariable Cox proportional hazard models.

#### Longitudinal analysis

Longitudinal data from each individual biomarker from baseline to 48 weeks were analyzed using mixed-effects modeling using maximum likelihood estimation of coefficients, with each log_10_-transformed biomarker, HIV status, age, edema, country, WHZ, presence of diarrhea, infection/sepsis, breastfeeding, and comorbidities being offered to the model as fixed effects, and participant identifier offered as the random effect. The fixed-effects covariates were selected using a theory-driven approach following a directed acyclic graph that included potential confounders, as shown in fig. S2. The longitudinal values from children with SAM were compared with the control values from a single sampling time point.

Missing outcome data affected a number of biomarkers and was assumed to have a Missing At Random (MAR) mechanism (38). To support this assumption and to produce unbiased inferences, the longitudinal adjusted mixed effect models had to include covariates that were predictive of the outcome being missing. Adaptive mixed effect lasso selection models were applied separately to an indicator for a biomarker representing if the record was missing or observed. The models explored which covariates predicted a biomarker record being missing and kept predictive covariates. The covariates indicated to be predictive of missingness were a subset of the fixed effects in the longitudinal models and thus further auxiliary variables were not required to be added to support the missing data assumption.

The robustness of the inferences to departure from the missing data assumption of MAR was explored through sensitivity analyses. Extreme values for the missing biomarker values were imputed using the 25^th^ and 75^th^ centile of the observed biomarker separately. The adjusted mixed effect models were then reapplied to the imputed data to explore if inferential changes could be observed for these extreme departures from MAR. The models demonstrated robustness to extreme departures and thus plausible departures would have not changed inferential conclusions.

Correction for multiple hypothesis testing was undertaken using the Romano-Wolf multiple hypothesis correction, and the adjusted P-value reported. Unadjusted results of longitudinal biomarkers, without offering any covariates, are shown in Fig. S7.

All statistical analysis was carried out using Stata (StataCorp. 2023. *Stata Statistical Software: Release 18*.)

## Supplementary Material

Supplementary Material

## Figures and Tables

**Table 1:** Baseline demographics of study participants, split by nutritional status and HIV status WHZ – weight-for-height z-score; MUAC – mid-upper arm circumference; HAZ – height-for-age z-score; IQR – inter-quarMle range; f/u – followup; ART – anMretroviral therapy. ^1^Co-morbidities and diagnoses were physician-diagnosed within 72h of admission. ^2^The P-values are from a Mann-Whitney U-test for continuous variables or a chi^2^ test for categorical variables, comparing children by HIV status within each nutritional group.

	Adequately-nourished children				Children with SAM				*P-value SAM vs control^2^*
Variable	Overall	HIV negative	HIV positive	*P-value^1^*	Overall	HIV negative	HIV positive	*P-value^1^*	
Total n (%)	173	113	60		264	204	60		
**Male, n (%)**	100	69 (61%)	31 (52%)	*0.234*	141	108 (53%)	33 (55%)	*0.779*	0.366
**Zimbabwe, n (%)**	103	70 (62%)	33 (55%)	*0.376*	198	161 (79%)	37 (62%)	*0.007*	0.001
**Age,** median, months; (IQR)	28	23	35	*0.009*	18	18	21	*0.025*	<0.001
	(18, 42)	(14, 40)	(22, 45)		(13, 23)	(13, 22)	(13, 27)		
**Oedema**, n (%)	-	-	-	*N/A*	177 (67%)	142 (70%)	35 (58%)	*0.092*	N/A
**WHZ** (median, IQR)	0.12 (-0.39, 0.84)	0.17 (-0.36, 0.96)	0.11 (-0.42, 0.53)	*0.852*	-2.87 (-3.98, -1.55)	-2.75 (-3.92, -1.41)	-3.51 (-4.64,-2.24)	*0.003*	<0.001
**MUAC** (cm, median (IQR))	15.0 (14.2, 16.1)	15.3 (14.5, 16.2)	14.9 (13.8, 15.9)	*0.055*	11.9 (11.0, 13.0)	12.0 (11.3, 13.4)	11.0 (10.0, 12.1)	*<0.001*	<0.001
**HAZ** (median, IQR)	-1.72 (-2.75, -0.76)	-1.39 (-2.55, -0.46)	-2.27 (-3.69,-1.28)	*<0.001*	-2.99 (-3.78, -1.94)	-2.89 (-3.70, -1.79)	-3.19 (-4.00,-2.24)	*0.047*	<0.001
- Severely stunted (HAZ < -3), n (%)	29 (17%)	12 (11%)	17 (28%)	*0.003*	128 (49%)	93 (46%)	35 (58%)	*0.082*	<0.001
**Birthweight**, kg, median (IQR)	3.01 (2.70, 3.40)	3.00 (2.60, 3.47)	3.03 (2.70, 3.40)	*0.917*	2.94 (2.50, 3.30)	2.90 (2.45, 3.13)	2.96 (2.50, 3.35)	*0.184*	0.016
**HIV-exposed uninfected, n (%)**	35 (20%)	35 (31%)	-	*N/A*	40 (15%)	40 (20%)	-	*N/A*	0.168
**On ART**			59 (98%)		-	-	24 (40%)	*N/A*	<0.001
**Residence:** Urban, n (%)	139 (80%)	91 (81%)	48 (80%)	*0.726*	172 (65%)	147 (72%)	33 (55%)	*0.027*	0.009
**Currently breastfed**	39 (22%)	32 (28%)	7 (12%)	*0.013*	39 (15%)	33 (16%)	6 (10%)	*0.311*	0.038
**Sepsis^1^**	-	-	-	-	11 (4%)	7 (3%)	4 (7%)	*0.270*	N/A
**Pneumonia^1^**	-	-	-	-	22 (8%)	14 (7%)	8 (13%)	*0.111*	N/A
**Diarrhea^1^**					128 (48%)	98 (48%)	30 (50%)	*0.814*	N/A
**Dehydration^1^**	-	-	-	-	44 (17%)	32 (16%)	12 (20%)	*0.431*	N/A
**Tuberculosis^1^**	-	-	-	-	46 (17%)	22 (11%)	24 (40%)	*<0.001*	N/A
**Dermatosis^1^**	-	-	-	-	45 (17%)	35 (17%)	10 (17%)	*0.929*	N/A
**Oral Thrush^1^**	-	-	-	-	40 (15%)	27 (13%)	13 (22%)	*0.109*	N/A
**Cerebral Palsy^1^**	-	-	-	-	6 (2%)	5 (2%)	1 (2%)	*0.720*	N/A
**Outcomes:**									
**Inpatient Deaths**	**-**	-	-	-	16 (6%)	10 (5%)	6 (10%)	*0.038*	**-**
**Outpatient deaths**	**-**	-	-	-	12	5	7	*0.003*	-
**Readmission**	**-**	-	-	-	26	18	8	*0.303*	-
**Death or readmission**	**-**	-	-	-	54	33	21	*0.001*	**-**

**Table 2:** Numerical results for the longitudinal analysis of the mixed effects modelling Differences in the log_10_ concentrations of biomarkers are shown. ^1^Results are adjusted for WHZ, edema, HIV, age, diarrhoea, infections [sepsis, urinary tract infection (UTI), pneumonia, tuberculosis (TB)], country, breastfeeding, cerebral palsy. ^2^Adjusted P-value displayed is the result of the Romano-Wolf correction for multiple hypothesis testing. Results are shown in bold where P < 0.05.

	Variable (log_10_)	Controls vs SAM^1^	Adj. P- value^2^	Change in biomarker over 1 year (SAM only)^1^	P - value
**Inflammation**	**IL-1β** [pg/mL]	**-0.14 [-0.19, -0.10]**	**<0.001**	**0.04 [0.01, 0.06]**	**<0.001**
	**Interferon-γ** [pg/mL]	**-0.13 [-0.18, -0.08]**	**<0.001**	**0.03 [0.00, 0.06]**	**0.030**
	**CCL3** [pg/mL]	**-0.07 [-0.12, -0.02]**	**0.016**	**-0.05 [-0.08, -0.01]**	**0.015**
	**CCL4** [pg/mL]	**-0.07 [-0.10, -0.04]**	**<0.001**	**-0.020 [-0.04, -0.00]**	**0.026**
	**sCD14** [pg/mL]	**-0.15 [-0.24, -0.06]**	**0.005**	-0.03 [-0.10, 0.03]	0.341
	**IL10** [pg/mL]	**-0.17 [-0.21, -0.12]**	**<0.001**	**-0.08 [-0.12, -0.04]**	**<0.001**
	**CRP** [mg/L]	-0.15 [-0.43, 0.13]	0.814	**-0.23 [-0.44, -0.02]**	**0.033**
	**LBP** [pg/mL]	**-0.25 [-0.38, -0.11]**	**0.002**	**-0.20 [-0.28, -0.12]**	**<0.001**
	**D-dimer** [pg/mL]	**0.19 [0.06, 0.32]**	**0.016**	-0.05 [-0.15, 0.06]	0.404
	**CD163** [ng/mL]	0.01 [-0.12, 0.14]	0.892	0.10 [-0.00, 0.21]	0.061
	**TNFa** [pg/mL]	**-0.07 [-0.11, -0.02]**	**0.011**	**0.03 [0.00, 0.06]**	**0.037**
	**IL-1ra** [pg/mL]	**-0.13 [-0.21, -0.05]**	**0.006**	**-0.15 [-0.21, -0.08]**	**<0.001**
	**IL-6** [pg/mL]	**-0.18 [-0.24, -0.11]**	**<0.001**	**-0.10 [-0.15, -0.05]**	**<0.001**
	**IL-33** [pg/mL]	**-0.09 [-0.13, -0.04]**	**0.002**	**0.07 [0.04, 0.10]**	**<0.001**
	**IL-8** [pg/mL]	**-0.18 [-0.26, -0.10]**	**0.001**	**-0.15 [-0.21, -0.08]**	**<0.001**
	**Eotaxin** [pg/mL]	**-0.34 [-0.43, -0.26]**	**<0.001**	0.04 [-0.01, 0.09]	0.087
	**IL-2** [pg/mL]	**-0.16 [-0.21, -0.10]**	**<0.001**	0.01 [-0.03, 0.05]	0.545
**Endothelial Activation**	**VCAM-1** [pg/mL]	**-0.47 [-0.61, -0.32]**	**<0.001**	-0.08 [-0.17, 0.02]	0.105
	**P-selection** [pg/mL]	**-0.15 [-0.20, -0.10]**	**<0.001**	0.00 [-0.04, 0.03]	0.804
	**L-selection** [pg/mL]	**-0.17 [-0.28, -0.05]**	**0.016**	**0.08 [0.03, 0.13]**	**0.002**
	**TPO** [pg/mL]	**-0.13 [-0.18, -0.09]**	**<0.001**	0.00 [-0.02, 0.03]	0.939
	**ICAM-1**[pg/mL]	-0.08 [-0.24, 0.08]	0.814	-0.02 [-0.06, 0.02]	0.238
**Growth Factors**	**VEGF** [pg/mL]	**-0.17 [-0.25, -0.09]**	**0.002**	**-0.08 [-0.13, -0.03]**	**0.0018**
	**EGF** [pg/mL]	-0.04 [-0.14, 0.05]	0.814	**0.15 [0.08, 0.22]**	**<0.001**
	**PlGF** [pg/mL]	-0.03 [-0.08, 0.03]	0.814	0.01 [-0.02, 0.05]	0.427
	**GM-CSF** [pg/mL]	**0.27 [0.03, 0.51]**	**0.048**	0.03 [-0.15, 0.21]	0.775
	**G-CSF** [pg/mL]	0.05 [-0.04, 0.15]	0.814	-0.01 [-0.09, 0.08]	0.863
	**Angiopoietion** [pg/mL]	**-0.20 [-0.33, -0.07]**	**0.013**	**-0.09 [-0.17, -0.00]**	**0.039**
	**IGFBP-3** [pg/mL]	**-0.33 [-0.48, -0.18]**	**0.002**	-0.02 [-0.13, 0.10]	0.792
**Enteropathy Biomarkers**	**A1AT** [mg/mL]	0.07 [-0.08, 0.23]	0.814	0.11 [-0.01, 0.23]	0.069
	**Neopterin** [nmol/L]	**-0.46 [-0.70, -0.23]**	**0.002**	**-0.59 [-0.77, -0.41]**	**<0.001**
	**MPO** [ng/mL]	0.15 [-0.22, 0.52]	0.814	0.20 [-0.08, 0.49]	0.160
	**GLP2** [ng/mL]	-0.12 [-0.34, 0.10]	0.814	**-1.15 [-1.32, -0.97]**	**<0.001**
	**I-FABP** [pg/mL]	-0.18 [-0.34, -0.01]	0.056	**-0.17 [-0.29, -0.06]**	**0.004**

**Table 3:** Individual baseline biomarkers of the three components associated with poor outcomes Individual baseline biomarkers of the Systemic Component 2, Systemic Component 3, Gut Component 1, and their associations with death or readmission. ^1^Adjusted for HIV, infections/sepsis (sepsis, pneumonia, TB, and UTI), baseline oedema, WHZ, and maternal education. Bold indicates P-value <0.05. 95%CI: 95% confidence interval. EGF - epidermal growth factor; VEGF - vascular endothelial growth factor; GCSF - granulocyte colony-stimulating factor; LBP - lipopolysaccharide binding protein; VCAM-1 - Vascular cell adhesion molecule 1; PlGF - placental growth factor; GLP2 - Glucagon-like peptide 2; I-FABP - intestinal fatty-acid binding protein; A1AT - alpha-1-antitrypsin.

		Univariable analysis		Multivariable analysis	
	Biomarker (log_10_ result)	Hazard Ratio [95%CI]	P-value	Adjusted hazard ratio^1^ [95%CI]	P-value
**Systemic Component 3**	**EGF**	**0.61 [0.39, 0.95]**	**0.028**	0.67 [0.42, 1.08]	0.101
	**VEGF**	**0.45 [0.26, 0.76]**	**<0.001**	**0.50 [0.28, 0.89]**	**0.019**
	**CCL3**	0.97 [0.47, 2.04]	0.946	1.04 [0.48, 2.23]	0.926
	**D-dimer**	1.16 [0.82, 1.63]	0.415	0.96 [0.65, 1.41]	0.820
	**P-selectin**	0.55 [0.23, 1.33]	0.185	0.53 [0.22, 1.28]	0.155
	**GCSF**	1.07 [0.71, 1.60]	0.761	0.95 [0.61, 1.49]	0.823
	**Angiopoietin**	**0.73 [0.54, 0.98]**	**0.038**	0.90 [0.62, 1.31]	0.583
**Systemic Component 2**	**sCD14**	0.80 [0.60, 1.07]	0.129	0.78 [0.58, 1.05]	0.102
	**LBP**	0.91 [0.68, 1.22]	0.540	0.87 [0.64, 1.16]	0.340
	**CD163**	0.92 [0.79, 1.07]	0.274	0.88 [0.76, 1.02]	0.080
	**L-selectin**	0.89 [0.58, 1.36]	0.587	0.77 [0.51, 1.17]	0.226
	**IL1-ra**	1.36 [0.88, 2.10]	0.170	1.15 [0.71, 1.86]	0.562
	**GM-CSF**	1.00 [0.88, 1.15]	0.956	0.94 [0.82, 1.08]	0.376
	**P-selectin**	0.55 [0.23, 1.33]	0.185	0.53 [0.22, 1.28]	0.155
	**VCAM-1**	1.20 [0.80, 1.81]	0.380	1.01 [0.68, 1.50]	0.942
	**PlGF**	1.05 [0.83, 1.34]	0.678	0.93 [0.71, 1.20]	0.564
**Gut Component 1**	**GLP2**	**0.81 [0.67, 0.99]**	**0.036**	**0.75 [0.61, 0.93]**	**0.010**
	**I-FABP**	**0.80 [0.65, 0.99]**	**0.045**	**0.77 [0.62, 0.97]**	**0.023**
	**Myeloperoxidase**	1.24 [0.98, 1.57]	0.073	1.15 [0.90, 1.45]	0.262
	**A1AT**	1.18 [0.83, 1.67]	0.361	1.41 [0.94, 2.12]	0.094

## Data Availability

All data associated with this study are present in the paper or supplementary materials.

## One Sentence Summary

Severe acute malnutrition is characterised by systemic, endothelial, and intestinal inflammation, which is exacerbated by HIV infection, persists over 48 weeks despite nutritional recovery, and is associated with death or hospital readmission.
