# Supplementary material for: Inflammation and epithelial repair predict mortality, hospital readmission and growth recovery in complicated severe acute malnutrition
Source: Sci Transl Med. Author manuscript; Available in PMC 2024 Apr 1. (PMC7615785; doi:10.1126/scitranslmed.adh0673)
Supplement: Supplementary Material [file EMS194379-supplement-Supplementary_Material.pdf]

Inflammation and epithelial repair predict mortality, hospital readmission and growth recovery in complicated severe acute malnutrition

Sturgeon et al.

Figure S1: Correlations between biomarkers at baseline 2
Figure S2: Directed acyclic graph (DAG) of potential confounders affecting the relationship between malnutrition and biomarker concentrations. 3
Figure S3: Principal Components Analysis showing changes in component loadings over time 4
Figure S4: Recovery of WHZ scores over time 5
Figure S5: Directed acyclic graph (DAG) examining confounders affecting biomarker concentrations and poor outcome 6
Figure S6: Example/Base model analysed using structural equation modelling 7
Figure S7: Longitudinal biomarkers from baseline to 48 weeks, unadjusted results 8

Table S1: Differences between the 264 children in the analysis, and the 15 children not included 9
Table S2: List of the biomarkers analysed 10
Table S3: Missingness of biospecimens and results at baseline 11
Table S4: Comparisons of biomarker concentrations across HIV and SAM groups 12
Table S5: Sensitivity analysis of longitudinal biomarker changes 13
Table S6: Differences between children who were able to provide a sample at 48-weeks, and those who did not 14
Table S7: Changes in concentrations of biomarkers in children with nutritional failure/relapse 15
Table S8: Missingness of biospecimens over the 48 weeks of the study 16

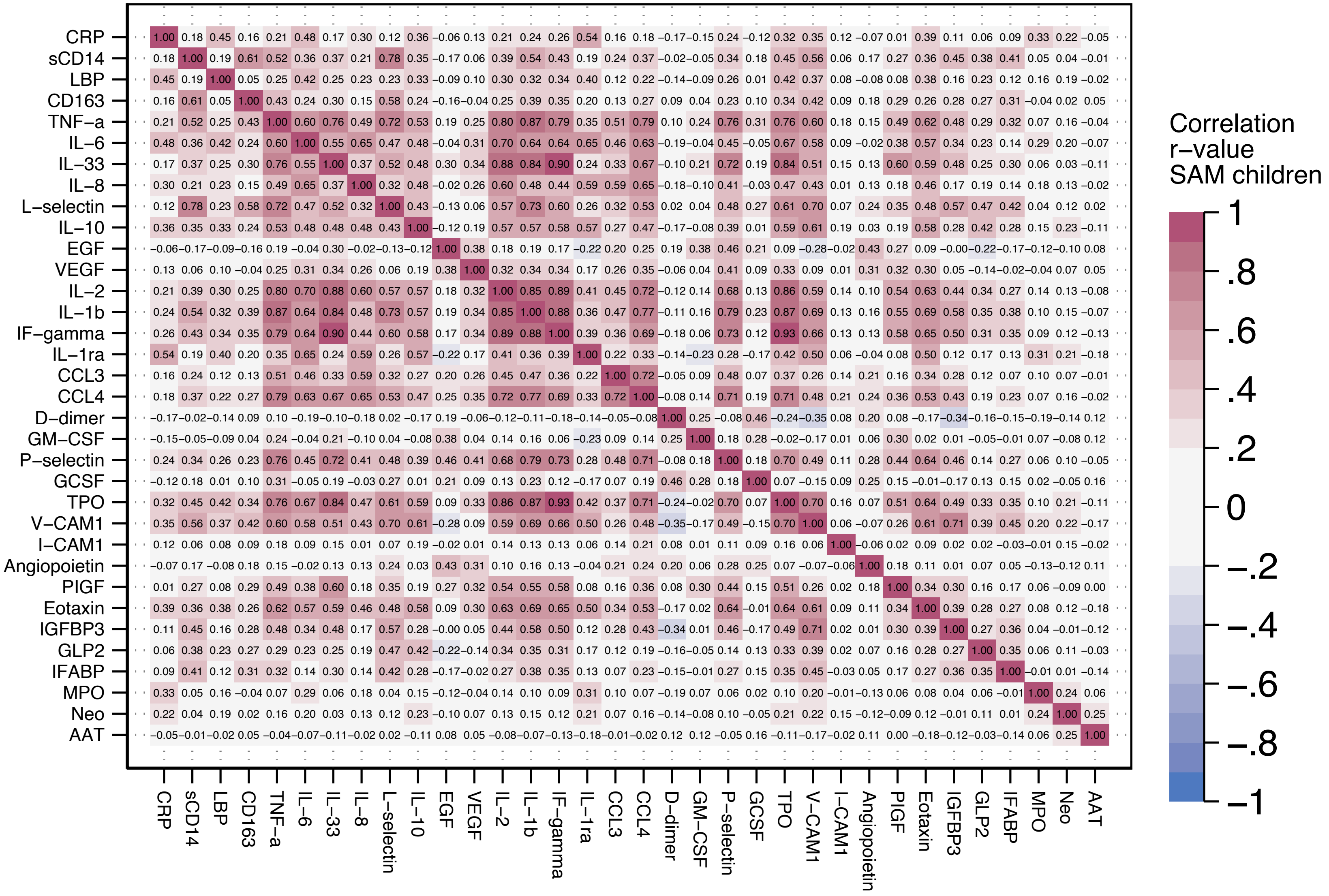

Figure S1: Correlations between biomarkers at baseline

Measured by Spearman's correlation coefficient of log_{10}-transformed biomarker.

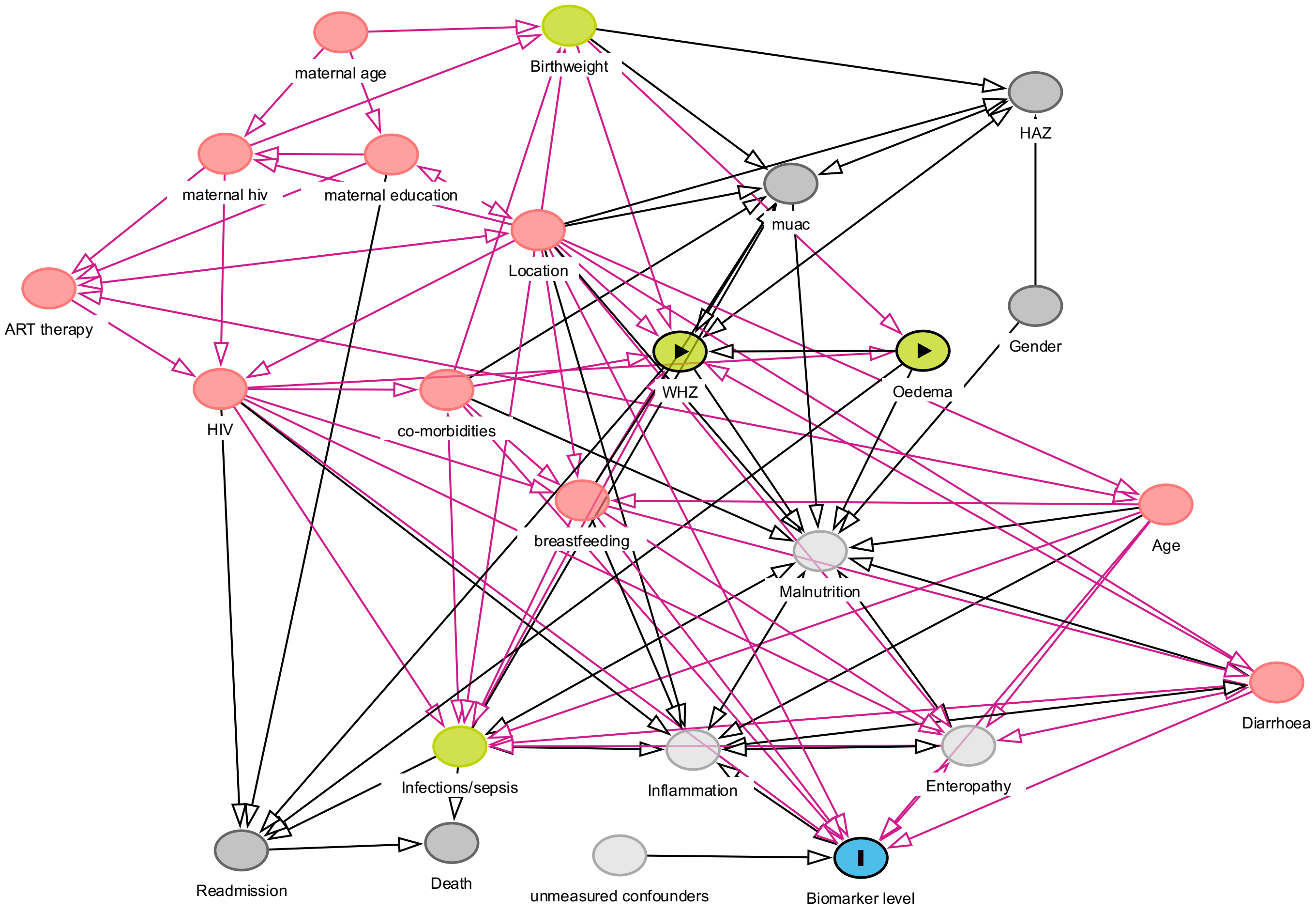

Figure S2: Directed acyclic graph (DAG) of potential confounders affecting the relationship between malnutrition and biomarker concentrations.

The minimum sufficient adjustment includes age, diarrhea, HIV, infections/sepsis, location, breastfeeding, and co-morbidities. Malnutrition was defined as low WHZ and/or baseline edema; both are included because severity and type of malnutrition may influence relationships in the DAG.

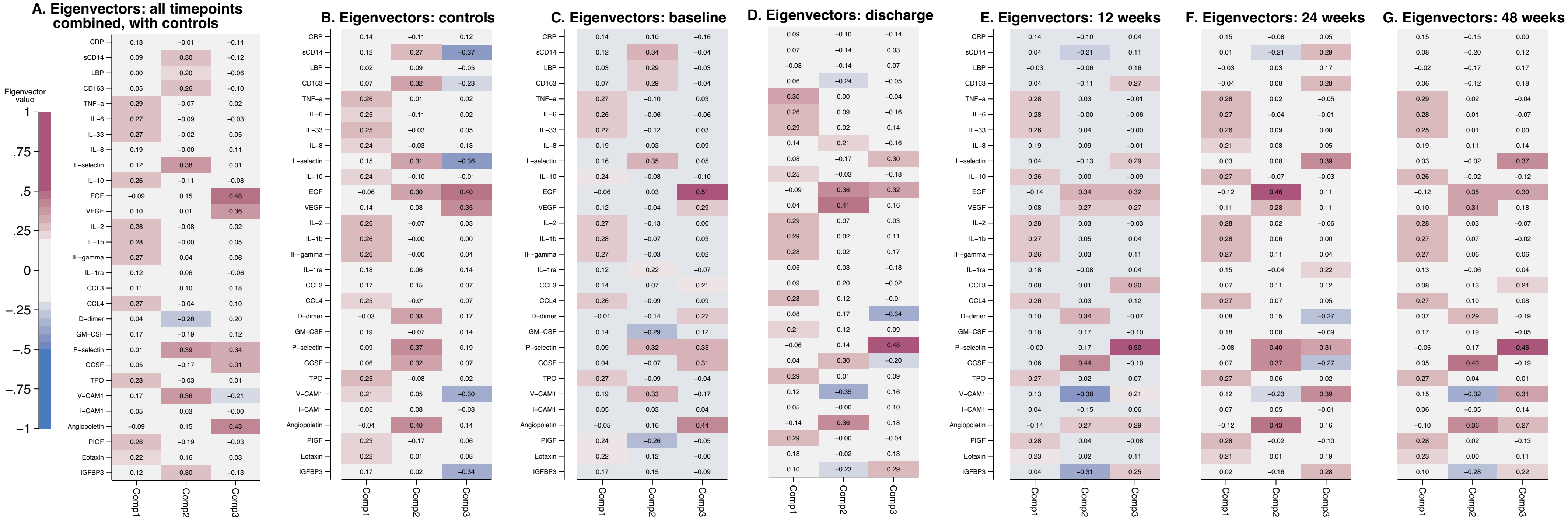

Figure S3: Principal Components Analysis showing changes in component loadings over time

These show the principal component analysis for different groups, including A) all timepoints combined with cases and controls, B) controls only C) SAM children at baseline, D) SAM children at discharge, E) SAM children at 12 weeks, F) SAM children at 24 weeks, and G) SAM children at 48 weeks.

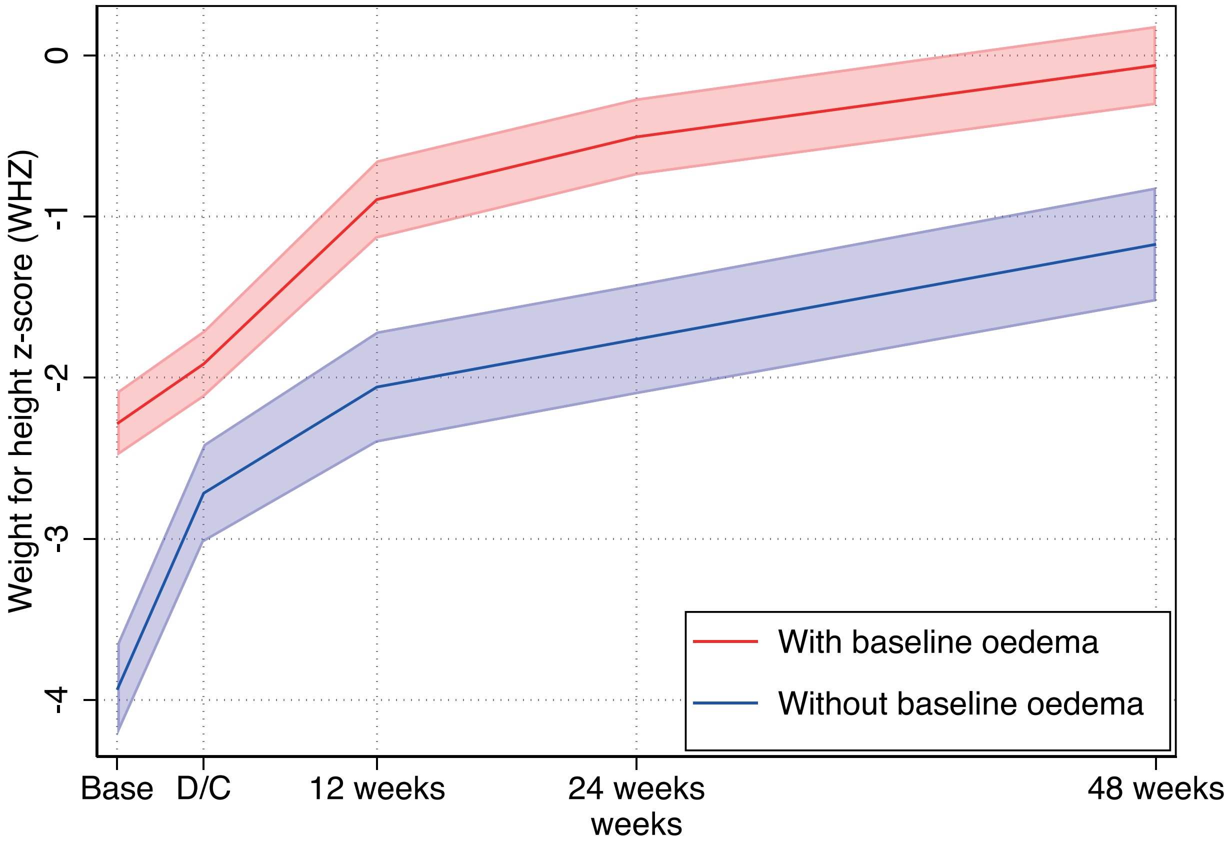

Figure S4: Recovery of WHZ scores over time

Children who had edema at baseline are shown in red, those without oedema are shown in blue.

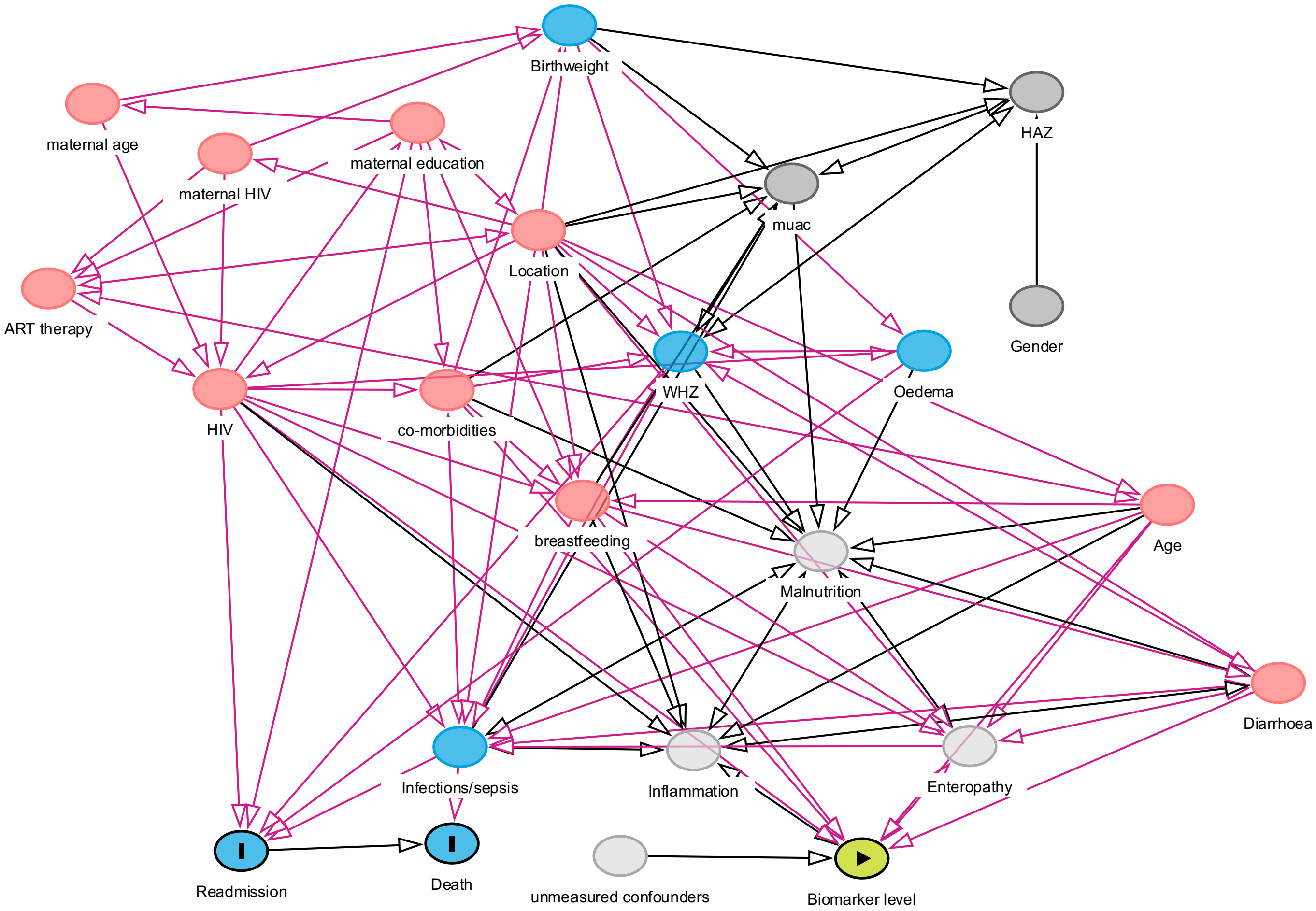

Figure S5: Directed acyclic graph (DAG) examining confounders affecting biomarker concentrations and poor outcome

Poor outcome was defined as a composite of death or readmission. The minimum sufficient adjustment set includes HIV, infections/sepsis, edema, WHZ, and maternal education.

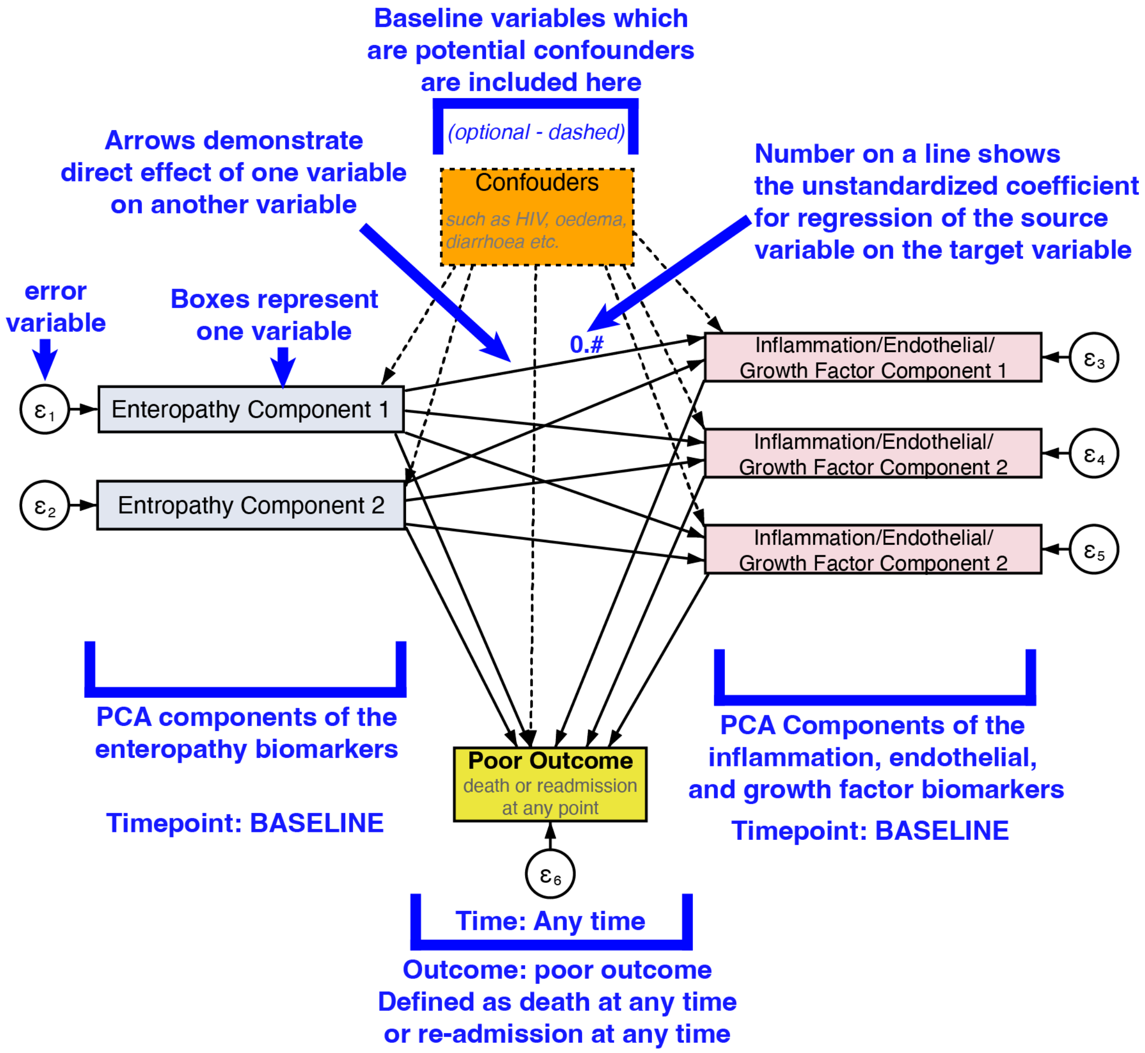

Figure S6: Example/Base model analysed using structural equation modelling

This shows the base model offered up to the structural equation modelling, which consisted of the two Gut Components (light blue), and the three Systemic Components (pink). Associations were tested with a composite 'poor outcome' which was defined as death or readmission at any point during the study.

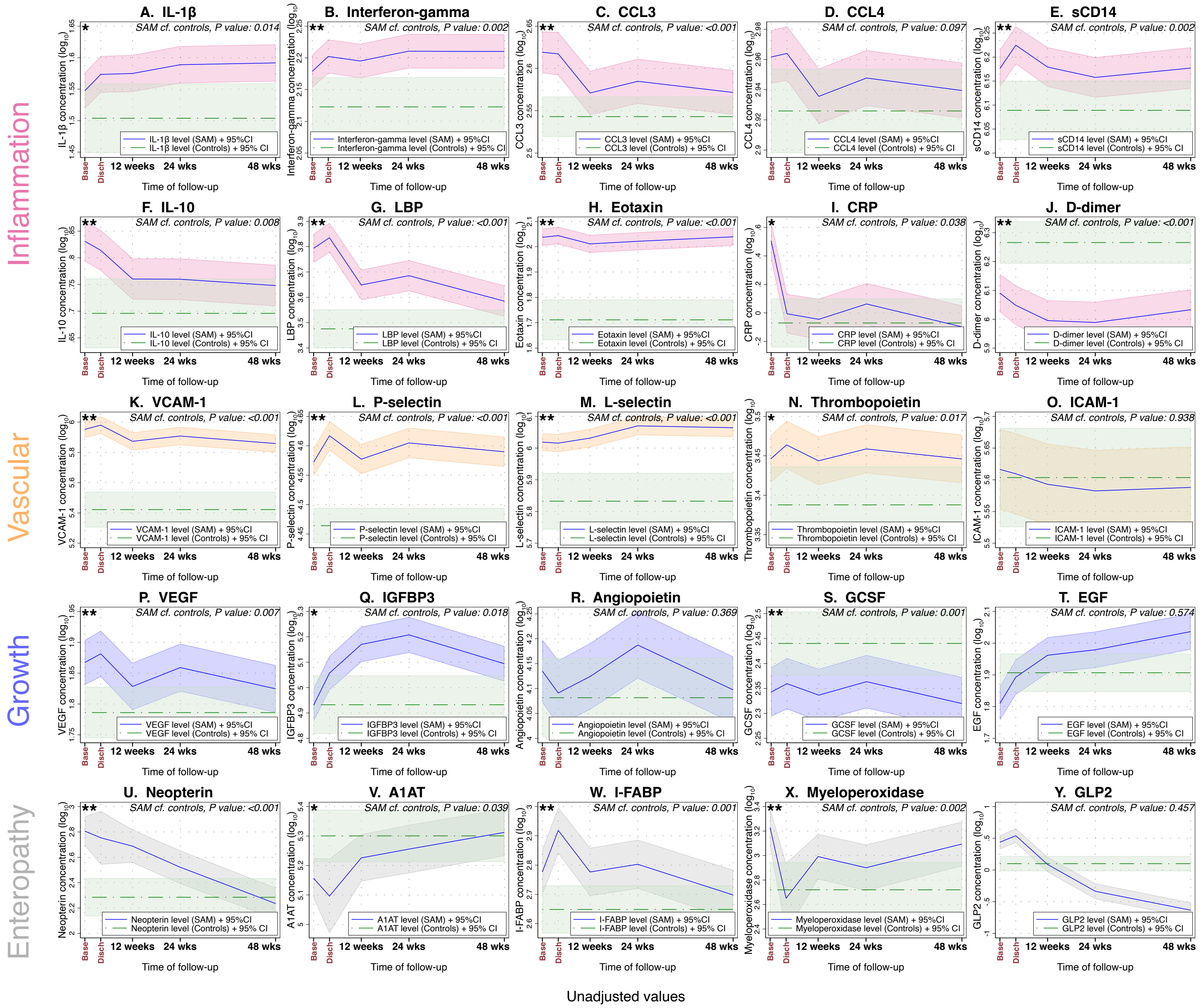

Figure S7: Longitudinal biomarkers from baseline to 48 weeks, unadjusted results

Biomarkers measured during inpatient stay at baseline and discharge (shown in red text on the graph), and at 12 weeks, 24 weeks, and 48 weeks after discharge. Biomarker concentrations are shown for systemic inflammation (pink): A) IL-1β, B) interferon-gamma, C) chemokine ligand 3 (CCL3), D) CCL4, E) soluble CD14, F) IL-10, G) lipopolysaccharide binding protein (LBP), H) eotaxin, I) C-reactive protein (CRP), and J) D-dimer. Vascular activation marker concentrations (yellow) shown include K) vascular cell adhesion molecule 1 (VCAM-1), L) P-selectin, M) L-selectin, N) thrombopoietin and O) intercellular adhesion molecule-1 (ICAM-1).. Growth factor concentrations (blue): P) vascular endothelial growth factor (VEGF), Q) insulin-like growth factor binding protein 3 (IGFBP3), R) angiopoietin, S) granulocyte-colony stimulating factor (GCSF), and T) epidermal growth factor (EGF). Enteropathy marker concentrations (grey): U) neopterin, V) alpha-1-antitrypsin (A1AT), W) intestinal fatty-acid binding protein (I-FABP), X) myeloperoxidase, and Y) glucagon-like peptide 2 (GLP2).

The results were analysed using mixed effects modelling, with the $\log_{10}$ transformed biomarker concentration and study week offered to the model as fixed effects, and participant identifier as the random effect. The P-value displayed is the value from this model for the difference between the longitudinal value for cases, and the baseline healthy value, and not adjusted for multiple hypothesis testing. ** = $P < 0.01$; * = $P < 0.05$; 95% CI – 95% confidence interval.

Table S1: Differences between the 264 children in the analysis, and the 15 children not included

Differences between the 264 children in the analysis, and the 15 who were intended to be in the study but did not provide any biospecimens, so were excluded from analysis. Children who did not provide a specimen were more likely to be from Zambia, had a smaller MUAC, were more likely to be HIV positive, to be from an urban environment and to have died (often the reason for missing samples). [1]The P-values are from a Mann-Whitney U-test for continuous variables or a chi^2 test for a categorical variable. WHZ – weight-for-height z-score; MUAC – mid-upper arm circumference; HAZ – height-for-age z-score; IQR – inter-quartile range; f/u – follow-up.

	SAM children			
**Variable**	**Total**	**Included in analysis**	**Not included in analysis**	***P-value*[1]**
**Total n**	**279**	**264**	**15**	
**Male**	**149**	**141**	**8**	0.995
**Zimbabwe**	**204**	**198**	**6**	**0.003**
Anthropometry				
**Age,** median, months; (IQR)	**18** (13, 23)	**18** (13, 23)	**17** (14, 21)	0.336
**Edema** (n)	**189**	**177**	**12**	0.634
**WHZ** (median, IQR)	**-2.98** (-3.98, -1.59)	**-2.87** (-3.98, -1.55)	**-3.14** (-4.01, -3.01)	0.181
**MUAC** (cm, median (IQR))	**11.8** (10.9, 13.0)	**11.9** (11.0, 13.0)	**10.6** (9.57, 11.4)	**0.001**
**HAZ** (median, IQR)	**-2.99** (-3.76, -2.00)	**-2.99** (-3.78, -1.94)	**-3.30** (-3.69, -2.81)	0.373
- Severe stunted (HAZ < -3)	**136**	**128**	8	0.715
**HIV positive**	**214**	**204**	**10**	**0.015**
**Birthweight** (kg, median)	**2.92** (2.50, 3.30)	**2.94** (2.50, 3.30)	**2.90** (2.40, 3.40)	
**HIV-exposed uninfected**	44	40	4	0.234
**Residence:** Urban	**187**	**172**	**15**	**<0.001**
**Outcomes:**				
Died during study	**40**	**28**	**12**	**<0.001**
During hospital admission:				
- Died as inpatient	**25**	**16**	9	***0.038***
- Discharged	**246**	**242**	4	*0.059*
- Withdrew/lost to follow-up in hospital	**8**	**6**	2	*0.913*
Readmitted during f/u	**30**	**26**	4	***0.032***

Table S2: List of the biomarkers analysed

The Luminex panel was one single 25-plex panel (Assay code: UgRYKY2M; R&D Systems). ELISAs were run separately.

Short name	Full name	Method
CRP	C-reactive protein	ELISA
sCD14	Soluble CD14	ELISA
LBP	Lipopolysaccharide-binding-protein	ELISA
sCD163	Soluble CD163	ELISA
TNFα	Tumour necrosis factor-alpha	Luminex panel
IL-6	Interleukin-6	Luminex panel
IL-33	Interleukin-33	Luminex panel
IL-8	Interleukin-8	Luminex panel
L-selectin	L-selectin	Luminex panel
IL-10	Interleukin-10	Luminex panel
EGF	Epidermal growth factor	Luminex panel
VEGF	Vascular endothelial growth factor	Luminex panel
IL-2	Interleukin-2	Luminex panel
IL-1β	Interleukin-1β	Luminex panel
IFN-γ	Interferon-gamma	Luminex panel
IL-1ra	Interleukin -1 receptor antagonist	Luminex panel
CCL3	Chemokine (C-C motif) ligand 3	Luminex panel
CCL4	Chemokine (C-C motif) ligand 4	Luminex panel
D-dimer	D-dimer	Luminex panel
GM-CSF	Granulocyte-macrophage colony-stimulating factor	Luminex panel
P-selectin	P-selectin	Luminex panel
GCSF	Granulocyte colony-stimulating factor	Luminex panel
TPO	Thrombopoietin	Luminex panel
VCAM-1	Vascular cell adhesion molecule 1	Luminex panel
ICAM-1	Intercellular adhesion molecule 1	Luminex panel
Angiopoietin	Angiopoietin-1	Luminex panel
PlGF	Placental growth factor	Luminex panel
Eotaxin	Eotaxin-1 (CCL11)	Luminex panel
IGFBP-3	IGF-binding protein 3	Luminex panel
GLP-2	Glucagon-like peptide 2	ELISA
I-FABP	Intestinal fatty-acid binding protein	ELISA
MPO	Myeloperoxidase	ELISA
Neopterin	Neopterin	ELISA
A1AT	Alpha-1-antitrypsin	ELISA

Table S3: Missingness of biospecimens and results at baseline

Patterns of biomarker missingness are shown. The total in the first column shows the number of children with that missingness profile. ‘1’ indicates the biomarker result was available, and ‘0’ indicates that there was no valid result available, predominantly due to stool or plasma specimens being unavailable. CRP – C reactive protein; LBP – lipopolysaccharide binding protein; GLP2 – glucagon-like peptide 2; MPO – myeloperoxidase; A1AT – alpha-1-antitrypsin; Neo – neopterin; I-FABP – intestinal fatty-acid binding protein.

Children with SAM at baseline									
**Totals**	**CRP/CD14**	**CD163**	**LBP**	**Luminex**	**GLP2**	**MPO**	**A1AT**	**Neo**	**I-FABP**
**93**	**1**	**1**	**1**	**1**	1	1	1	1	1
**43**	**1**	**1**	**1**	**1**	1	0	0	0	1
35	0	0	0	0	0	0	0	0	0
18	0	0	0	0	0	1	1	1	0
**13**	**1**	**1**	**1**	**1**	0	0	0	0	1
13	1	1	1	0	1	1	1	1	1
**7**	**1**	**1**	**1**	**1**	0	1	1	0	1
**6**	**1**	**1**	**1**	**1**	1	1	1	0	1
6	1	1	1	0	1	0	0	0	1
5	0	0	0	1	0	0	0	0	0
**2**	**1**	**1**	**1**	**1**	1	1	0	0	1
**2**	**1**	**1**	**1**	**1**	0	0	0	1	1
2	1	1	1	1	1	1	1	0	1
2	1	1	1	0	0	1	1	0	1
2	1	1	1	0	0	0	0	0	1
**1**	**1**	**1**	**1**	**1**	1	0	0	1	1
**1**	**1**	**1**	**1**	**1**	0	1	1	1	1
**1**	**1**	**1**	**1**	**1**	0	0	0	0	0
1	1	1	1	0	1	0	0	1	0
1	1	1	1	0	0	1	1	1	0
1	1	1	1	0	0	0	0	1	1
1	1	1	0	1	1	1	1	0	1
1	1	1	0	0	1	1	1	0	1
1	1	0	0	1	1	0	0	0	0
1	1	0	0	1	0	0	0	0	1
1	0	1	0	1	0	0	0	0	0
1	0	0	0	1	1	0	0	0	1
1	0	0	0	0	0	1	1	0	0
1	0	0	0	0	0	1	0	1	0
1	0	0	0	0	0	0	0	1	0
	**Present = 1; Missing = 0**								
**264**	**201**	**200**	**197**	**181**	**171**	**149**	**146**	**133**	**198**
	88%	87%	86%	79%	75%	65%	64%	58%	86%
	**Total complete inflammatory/endothelial/growth - 169**				**Total complete enteropathy - 106**				

Table S4: Comparisons of biomarker concentrations across HIV and SAM groups

Comparisons between groups was done by OLS regression if biomarker concentration differed by 1/3 of the standard deviation of the baseline group (HIV- SAM- if present, or the largest group if not). [1]P-value was adjusted by using the Romano-Wolf multiple hypothesis correction; blank P-value indicated comparison not calculated. Result is bolded if the adjusted P-value <0.05. ANC – adequately-nourished controls. The full abbreviations for each biomarker are shown in Table S2.

Biomarker (mean $\log_{10}$, ± SD)	HIV negative		[1]Adj. P-value	HIV positive		[1]Adj. P-value	Adequately Nourished		[1]Adj. P-value	SAM		[1]Adj. P-value
	SAM+ve	SAM-ve		SAM+ve	SAM-ve		HIV-	HIV+		HIV-	HIV+	
C-reactive protein (mg/L)	**0.376 ±0.918**	**-0.132 ±1.121**	**0.008**	**0.833 ±0.931**	**0.045 ±1.120**	**0.009**	-0.132 ±1.12	0.045 ±1.120		0.376 ±0.918	0.833 ±0.931	0.081
CD14 (pg/mL)	6.160 ±0.327	6.071 ±0.445		6.228 ±0.411	6.122 ±0.308	0.211	6.071 ±0.445	6.122 ±0.308		6.160 ±0.327	6.228 ±0.411	
LBP (ng/mL)	**3.789 ±0.446**	**3.514 ±0.452**	**0.003**	**3.876 ±0.416**	**3.408 ±0.533**	**0.001**	3.514 ±0.452	3.408 ±0.533		3.789 ±0.446	3.876 ±0.416	
CD163 (ng/mL)	2.956 ±0.678	2.947 ±0.454		3.061 ±0.746	3.003 ±0.243		2.947 ±0.454	3.003 ±0.243		2.956 ±0.678	3.061 ±0.746	
TNF-α (pg/mL)	1.478 ±0.273	1.502 ±0.333		1.649 ±0.301	1.516 ±0.397		1.502 ±0.333	1.516 ±0.397		**1.478 ±0.273**	**1.649 ±0.301**	**0.028**
IL6 (pg/mL)	1.114 ±0.382	1.009 ±0.423		**1.344 ±0.291**	**1.046 ±0.514**	**0.037**	1.009 ±0.423	1.046 ±0.514		**1.114 ±0.382**	**1.344 ±0.291**	**0.017**
IL-33 (pg/mL)	1.861 ±0.226	1.901 ±0.289		1.959 ±0.273	1.861 ±0.348		1.901 ±0.289	1.861 ±0.348		1.861 ±0.226	1.959 ±0.273	0.140
IL-8 (pg/mL)	**1.529 ±0.421**	**1.296 ±0.298**	**0.003**	1.589 ±0.345	1.343 ±0.409	0.059	1.296 ±0.298	1.343 ±0.409		1.529 ±0.421	1.589 ±0.345	
L-selectin (pg/mL)	6.010 ±0.303	5.835 ±0.627		6.048 ±0.170	5.828 ±0.449	0.077	5.835 ±0.627	5.828 ±0.449		6.010 ±0.303	6.048 ±0.170	
IL-10 (pg/mL)	0.816 ±0.263	0.694 ±0.397		0.925 ±0.318	0.699 ±0.460	0.109	0.694 ±0.397	0.699 ±0.460		0.816 ±0.263	0.925 ±0.318	0.167
EGF (pg/mL)	1.845 ±0.370	1.874 ±0.371		**1.669 ±0.368**	**1.970 ±0.409**	**0.011**	1.874 ±0.371	1.970 ±0.409		1.845 ±0.370	1.669 ±0.368	0.109
VEGF (pg/mL)	1.894 ±0.266	1.790 ±0.241	0.071	1.807 ±0.340	1.779 ±0.315		1.790 ±0.241	1.779 ±0.315		1.894 ±0.266	1.807 ±0.340	
IL-2 (pg/mL)	1.610 ±0.306	1.580 ±0.398		1.746 ±0.363	1.577 ±0.481	0.210	1.580 ±0.398	1.577 ±0.481		1.610 ±0.306	1.746 ±0.363	0.124
IL-1β (pg/mL)	1.536 ±0.243	1.506 ±0.324		1.610 ±0.281	1.500 ±0.406		1.506 ±0.324	1.500 ±0.406		1.536 ±0.243	1.610 ±0.281	
Interferon-γ (pg/mL)	2.162 ±0.218	2.132 ±0.268		2.245 ±0.232	2.105 ±0.353	0.161	2.132 ±0.268	2.105 ±0.353		2.162 ±0.218	2.245 ±0.232	0.181
IL-1ra (pg/mL)	**3.107 ±0.322**	**2.811 ±0.222**	**0.001**	**3.319 ±0.304**	**2.872 ±0.313**	**0.001**	2.811 ±0.222	2.872 ±0.313		**3.107 ±0.322**	**3.319 ±0.304**	**0.011**
CCL3 (pg/mL)	2.626 ±0.214	2.551 ±0.161	0.081	2.607 ±0.161	2.529 ±0.135	0.121	2.551 ±0.161	2.529 ±0.135		2.626 ±0.214	2.607 ±0.161	
CCL4 (pg/mL)	2.958 ±0.158	2.927 ±0.161		2.991 ±0.145	2.924 ±0.210		2.927 ±0.161	2.924 ±0.210		2.958 ±0.158	2.991 ±0.145	
D-dimer (pg/mL)	6.066 ±0.427	6.226 ±0.479	0.109	6.176 ±0.424	6.342 ±0.450	0.211	6.226 ±0.479	6.342 ±0.450		6.066 ±0.427	6.176 ±0.424	
IGFBP-3 (pg/mL)	4.928 ±0.455	4.976 ±0.738		4.948 ±0.415	4.849 ±0.740		4.976 ±0.738	4.849 ±0.740		4.928 ±0.455	4.948 ±0.415	
GM-CSF (pg/mL)	**0.527 ±1.088**	**1.077 ±0.660**	**0.003**	0.885 ±1.131	1.251 ±0.552	0.181	1.077 ±0.660	1.251 ±0.552		0.527 ±1.088	0.885 ±1.131	
P-selectin (pg/mL)	**4.570 ±0.177**	**4.467 ±0.187**	**0.004**	4.575 ±0.165	4.460 ±0.202	0.077	4.467 ±0.187	4.460 ±0.202		4.570 ±0.177	4.575 ±0.165	
GCSF (pg/mL)	2.361 ±0.388	2.454 ±0.414		2.288 ±0.289	2.415 ±0.406		2.454 ±0.414	2.415 ±0.406		2.361 ±0.388	2.288 ±0.289	
Thrombopoietin (pg/mL)	3.424 ±0.222	3.391 ±0.286		3.526 ±0.256	3.381 ±0.359	0.167	3.391 ±0.286	3.381 ±0.359		3.424 ±0.222	3.526 ±0.256	0.121
VCAM-1 (pg/mL)	**5.911 ±0.457**	**5.442 ±0.731**	**0.001**	**6.110 ±0.346**	**5.384 ±0.805**	**0.001**	5.442 ±0.731	5.384 ±0.805		5.911 ±0.457	6.110 ±0.346	0.121
ICAM-1 (pg/mL)	5.595 ±0.499	5.620 ±0.481		5.708 ±0.304	5.573 ±0.542		5.620 ±0.481	5.573 ±0.542		**5.595 ±0.499**	**5.708 ±0.304**	**0.003**
Angiopoietin (pg/mL)	4.224 ±0.428	4.061 ±0.567		3.852 ±0.559	4.120 ±0.390	0.109	4.061 ±0.567	4.120 ±0.390		4.224 ±0.428	3.852 ±0.559	
PlGF (pg/mL)	0.947 ±0.555	1.117 ±0.596		1.211 ±0.694	1.180 ±0.697		1.117 ±0.596	1.180 ±0.697		0.947 ±0.555	1.211 ±0.694	0.121
Eotaxin (pg/mL)	**2.014 ±0.266**	**1.700 ±0.503**	**0.001**	**2.125 ±0.245**	**1.733 ±0.533**	**0.004**	1.700 ±0.503	1.733 ±0.533		2.014 ±0.266	2.125 ±0.245	0.128
GLP-2 (ng/mL)	**0.436 ±0.636**	**0.080 ±0.728**	**0.016**	0.518 ±0.605	0.132 ±0.599	0.089	0.080 ±0.728	0.132 ±0.599		0.436 ±0.636	0.518 ±0.605	
IFABP (pg/mL)	2.795 ±0.540	2.691 ±0.537		2.670 ±0.745	2.570 ±0.537		2.691 ±0.537	2.570 ±0.537		2.795 ±0.540	2.670 ±0.745	
MPO (ng/mL)	3.143 ±1.009	2.675 ±1.193	0.104	**3.532 ±0.645**	**2.815 ±1.108**	**0.049**	2.675 ±1.193	2.815 ±1.108		3.143 ±1.009	3.532 ±0.645	0.181
Neopterin (nmol/L)	**2.830 ±0.524**	**2.241 ±0.910**	**0.001**	2.783 ±0.936	2.379 ±0.784	0.211	2.241 ±0.910	2.379 ±0.784		2.830 ±0.524	2.783 ±0.936	
AAT (mg/mL)	5.147 ±0.474	5.284 ±0.475		5.211 ±0.428	5.334 ±0.409		5.284 ±0.475	5.334 ±0.409		5.147 ±0.474	5.211 ±0.428	
Number (n)	204	113		60	60		113	60		204	60	

Table S5: Sensitivity analysis of longitudinal biomarker changes

Longitudinal analyses were first re-run using a mixed effects model, with missing data replaced with high [75th centile of present values] or low [25th centile of present values] values, likely to represent extremes of results. The actual result was analysed to see if it fell within the lower and higher values. Only two biomarkers (IL-10 and IL-6) have been highlighted as potentially affected by missing data.
[1]Results are adjusted for WHZ, oedema, HIV age, diarrhoea, infections [sepsis, UTI, pneumonia, TB], country, breastfeeding, and cerebral palsy. The full abbreviations for each biomarker are shown in Table S2

		Original result +95%CI	1. Missing data replaced with high and low values compared with the data present +95% confidence interval		
	Variable (log_{10})	Controls vs SAM children[1]	With missing values set at 25th centile	With missing values set at 75th centile	Different effect due to missing data?
Inflammation	**IL-1β** [pg/mL]	**-0.14 [-0.19, -0.10]**	-0.13 [-0.18, -0.07]	-0.16 [-0.21, -0.12]	No
	**Interferon-γ** [pg/mL]	**-0.13 [-0.18, -0.08]**	-0.11 [-0.16, -0.06]	-0.16 [-0.20, -0.11]	No
	**CCL3** [pg/mL]	**-0.07 [-0.12, -0.02]**	-0.05 [-0.09, -0.01]	-0.07 [-0.12, -0.03]	No
	**CCL4** [pg/mL]	**-0.07 [-0.10, -0.04]**	-0.06 [-0.09, -0.03]	-0.09 [-0.11, -0.06]	No
	**CD14** [pg/mL]	**-0.15 [-0.24, -0.06]**	-0.13 [-0.21, -0.05]	-0.16 [-0.24, -0.08]	No
	**IL10** [pg/mL]	**-0.17 [-0.21, -0.12]**	-0.14 [-0.20, -0.08]	-0.22 [-0.28, -0.17]	Yes
	**CRP** [mg/L]	-0.15 [-0.43, 0.13]	-0.10 [-0.36, 0.16]	-0.24 [-0.49, 0.02]	No
	**LBP** [pg/mL]	**-0.25 [-0.38, -0.11]**	-0.20 [-0.31, -0.09]	-0.23 [-0.35, -0.12]	No
	**D-dimer** [pg/mL]	**0.19 [0.06, 0.32]**	0.22 [0.10, 0.33]	0.10 [-0.02, 0.21]	No
	**CD163** [ng/mL]	0.01 [-0.12, 0.14]	0.01 [-0.11, 0.13]	-0.02 [-0.14, 0.10]	No
	**TNF-α** [pg/mL]	**-0.07 [-0.11, -0.02]**	-0.06 [-0.12, -0.01]	-0.11 [-0.16, -0.07]	No
	**IL-1ra** [pg/mL]	**-0.13 [-0.21, -0.05]**	-0.10 [-0.17, -0.03]	-0.16 [-0.22, -0.09]	No
	**IL-6** [pg/mL]	**-0.18 [-0.24, -0.11]**	-0.14 [-0.22, -0.07]	-0.25 [-0.32, -0.18]	Yes
	**IL-33** [pg/mL]	**-0.09 [-0.13, -0.04]**	-0.07 [-0.12, -0.02]	-0.12 [-0.17, -0.08]	No
	**IL-8** [pg/mL]	**-0.18 [-0.26, -0.10]**	-0.14 [-0.21, -0.07]	-0.20 [-0.27, -0.13]	No
	**Eotaxin** [pg/mL]	**-0.34 [-0.43, -0.26]**	-0.30 [-0.39, -0.22]	-0.35 [-0.43, -0.27]	No
	**IL-2** [pg/mL]	**-0.16 [-0.21, -0.10]**	-0.13 [-0.20, -0.07]	-0.19 [-0.25, -0.14]	No
Endothelial Activation	**V-CAM1** [pg/mL]	**-0.47 [-0.61, -0.32]**	-0.42 [-0.55, -0.30]	-0.48 [-0.61, -0.35]	No
	**P-selectin** [pg/mL]	**-0.15 [-0.20, -0.10]**	-0.11 [-0.15, -0.06]	-0.15 [-0.19, -0.10]	No
	**L-selectin** [pg/mL]	**-0.17 [-0.28, -0.05]**	-0.14 [-0.25, -0.04]	-0.17 [-0.27, -0.06]	No
	**TPO** [pg/mL]	**-0.13 [-0.18, -0.09]**	-0.11 [-0.16, -0.07]	-0.16 [-0.20, -0.12]	No
	**I-CAM1**[pg/mL]	-0.08 [-0.24, 0.08]	-0.01 [-0.15, 0.12]	-0.08 [-0.21, 0.06]	No
Growth Factors	**VEGF** [pg/mL]	**-0.17 [-0.25, -0.09]**	-0.13 [-0.20, -0.06]	-0.18 [-0.25, -0.11]	No
	**EGF** [pg/mL]	-0.04 [-0.14, 0.05]	0.04 [-0.05, 0.13]	-0.06 [-0.15, 0.04]	No
	**PIGF** [pg/mL]	-0.03 [-0.08, 0.03]	-0.03 [-0.13, 0.07]	-0.08 [-0.16, 0.01]	No
	**GM-CSF** [pg/mL]	**0.27 [0.03, 0.51]**	0.21 [-0.01, 0.42]	0.08 [-0.14, 0.30]	No
	**G-CSF** [pg/mL]	0.05 [-0.04, 0.15]	0.09 [0.00, 0.18]	0.02 [-0.07, 0.10]	No
	**Angiopoietin** [pg/mL]	**-0.20 [-0.33, -0.07]**	-0.09 [-0.20, 0.02]	-0.19 [-0.31, -0.07]	No
	**IGFBP-3** [pg/mL]	**-0.33 [-0.48, -0.18]**	-0.25 [-0.38, -0.12]	-0.35 [-0.48, -0.23]	No
Enteropathy Biomarkers	**AAT** [mg/mL]	0.07 [-0.08, 0.23]	0.11 [0.02, 0.19]	0.01 [-0.08, 0.10]	No
	**Neopterin** [nmol/L]	**-0.46 [-0.70, -0.23]**	-0.23 [-0.37, -0.10]	-0.55 [-0.71, -0.39]	No
	**MPO** [ng/mL]	0.15 [-0.22, 0.52]	0.02 [-0.18, 0.22]	-0.10 [-0.32, 0.11]	No
	**GLP-2** [ng/mL]	-0.12 [-0.34, 0.10]	-0.11 [-0.28, 0.07]	-0.19 [-0.36, -0.02]	No
	**I-FABP** [pg/mL]	-0.18 [-0.34, -0.01]	-0.14 [-0.29, 0.01]	-0.20 [-0.35, -0.04]	No

Table S6: Differences between children who were able to provide a sample at 48-weeks, and those who did not

Baseline demographics of study participants, split by children who had an available sample at 48 weeks, and those who do not. WHZ – weight-for-height z-score; MUAC – mid-upper arm circumference; HAZ – height-for-age z-score; IQR – inter-quartile range; f/u – follow-up. 1Co-morbidities and diagnoses were physician diagnosed within 72h of admission. 2The P-values are from a Mann-Whitney U-test for continuous variables or a chi2 test for categorical variables.

	**Total n (%)**	**Those with 48 wk sample**	**No 48 wk sample**	***P-value*[2]**
**Total n (%)**	264	175	89	
**Male, n (%)**	141	99	42	*0.149*
**Zimbabwe, n (%)**	198	136	39	*0.153*
**Age,** median, months; (IQR)	18 (13, 23)	19 (14, 23)	18 (12, 22)	*0.157*
**Edema**, n (%)	177 (67%)	118	59	*0.95*
**WHZ** (median, IQR)	-2.87 (-3.98, -1.55)	-2.82 (-3.80, -1.61)	-2.91 (-4.13, -1.49)	*0.606*
**MUAC** (cm, median (IQR))	11.9 (11.0, 13.0)	12.0 (11.1, 13.1)	11.7 (10.5, 13.0)	*0.147*
**HAZ** (median, IQR)	-2.99 (-3.78, -1.94)	-3.01 (-3.78, -2.06)	-2.84 (-3.77, -1.70)	*0.278*
- Severely stunted (HAZ < -3), n (%)	128 (49%)			
**Birthweight**, kg, median (IQR)	2.94 (2.50, 3.30)	3.00 (2.50, 3.36)	2.82 (2.50, 3.14)	*0.200*
**HIV-exposed uninfected, n (%)**	40 (15.2%)	28 (16.0%)	12 (13.5%)	*0.590*
**HIV+, n(%)**	60	38 (21.7%)	22 (24.7%)	*0.582*
**On ART (if HIV+)**	-	14 (36.8%)	10 (45.4%)	*0.512*
**Residence:** Urban, n (%)	172 (65%)	120 (68.6%)	60 (68.2%)	*0.696*
**Currently breastfed**	39 (14.7%)	25 (14.3%)	14 (20.9%)	*0.211*
**Sepsis[1]**	11 (4.2%)	9 (5.1%)	2 (2.3%)	*0.266*
**Pneumonia[1]**	22 (8.3%)	11 (6.3%)	11 (12.4%)	*0.091*
**Diarrhea[1]**	128 (48.5%)	87 (49.7%)	41 (46.6%)	*0.633*
**Dehydration[1]**	44 (16.7%)	34 (19.4%)	10 (11.2%)	*0.091*
**Tuberculosis[1]**	46 (17.4%)	30 (17.1%)	16 (18.0%)	*0.866*
**Dermatosis[1]**	45 (17.0%)	31 (17.7%)	14 (15.7%)	*0.685*
**Oral Thrush[1]**	40 (15.2%)	26 (14.8%)	14 (15.7%)	*0.852*
**Cerebral Palsy[1]**	6 (2.3%)	5 (2.9%)	1 (1.1%)	*0.372*

Table S7: Changes in concentrations of biomarkers in children with nutritional failure/relapse

This table shows the log_{10} biomarker results for those children who had a nutritional relapse or failure at any point (defined as a having worse category of malnutrition than at a previous timepoint, or dying). [1]P-values were corrected for multiple hypothesis testing using the Romano-Wolf method. The full abbreviations for each biomarker are shown in Table S2.

**Biomarker** (log_{10} level)	**Nutritional failure/relapse**	**Corr P-Value***
**IL1b**	-0.01 [-0.05, 0.03]	0.911
**CD163**	-0.06 [-0.16, 0.04]	0.475
**TNFa**	0.00 [-0.04, 0.03]	1.000
**IL-1ra**	0.02 [-0.04, 0.09]	0.743
**IL6**	0.05 [-0.00, 0.10]	0.099
**IL33**	0.01 [-0.03, 0.04]	1.000
**IL8**	0.05 [-0.01, 0.11]	0.158
**Eotaxin**	-0.04 [-0.11, 0.03]	0.703
**IL-2**	0.03 [-0.01, 0.07]	0.495
**VCAM1**	-0.03 [-0.15, 0.09]	1.000
**P-selectin**	-0.02 [-0.06, 0.02]	0.891
**IFG**	0.00 [-0.04, 0.04]	1.000
**L-selectin**	-0.02 [-0.12, 0.07]	1.000
**TPO**	0.01 [-0.03, 0.04]	1.000
**I-CAM1**	0.05 [-0.10, 0.19]	1.000
**VEGF**	-0.01 [-0.07, 0.06]	1.000
**EGF**	0.00 [-0.08, 0.08]	1.000
**PIGF**	-0.01 [-0.05, 0.04]	1.000
**GM-CSF**	0.17 [-0.03, 0.36]	0.327
**G-CSF**	0.04 [-0.04, 0.11]	0.822
**Angiopoietin-1**	0.00 [-0.11, 0.10]	1.000
**IGFBP-3**	**-0.22 [-0.33, -0.10]**	**0.010**
**CCL3**	0.01 [-0.04, 0.05]	1.000
**AAT**	0.02 [-0.11, 0.15]	1.000
**Neo**	0.09 [-0.11, 0.30]	0.158
**MPO**	-0.07 [-0.37, 0.23]	0.980
**GLP2**	-0.20 [-0.37, -0.03]	0.921
**IFABP**	0.01 [-0.12, 0.13]	1.000
**CCL4**	0.01 [-0.01, 0.03]	0.475
**CD14**	-0.07 [-0.14, -0.00]	0.158
**IL10**	0.02 [-0.02, 0.06]	0.406
**CRP**	-0.07 [-0.29, 0.14]	1.000
**LBP**	-0.01 [-0.12, 0.10]	1.000
**D-dimer**	0.11 [0.01, 0.22]	0.089

Table S8: Missingness of biospecimens over the 48 weeks of the study

The missingness of the biomarkers over the course of the study. [1]This includes children who attended, as well as children who died (which were not considered missing). [2]This includes children who missed a follow-up visit, children who had formally withdrawn from the study, and children with no result because they were unable to provide a sample and/or the sample was insufficient for analysis.[3]Full biomarkers analysed by Luminex shown in Table S2.

		Number present at each timepoint					
**Marker**		**Baseline**	**Discharge**	**12 weeks**	**24 weeks**	**48 weeks**	**Average results per child for each marker**
**Luminex[3] (plasma)**	Present/died[1]	197	185	183	178	184	3.4
	Missed visit/ withdrew/insufficient[2]	67	79	81	86	80	
**CRP/CD14 (plasma)**	Present/died[1]	216	214	200	194	189	3.6
	Missed visit/ withdrew/insufficient[2]	48	50	64	70	75	
**CD163 (plasma)**	Present/died[1]	212	205	196	188	187	3.6
	Missed visit/ withdrew/insufficient[2]	52	59	68	76	77	
**IL-6 (plasma)**	Present/died[1]	197	185	183	178	184	3.4
	Missed visit/ withdrew/insufficient[2]	67	79	81	86	80	
**GLP2 (plasma)**	Present/died[1]	187	182	166	157	166	3.6
	Missed visit/ withdrew/insufficient[2]	77	82	98	107	98	
**I-FABP (plasma)**	Present/died[1]	213	212	201	193	189	3.6
	Missed visit/ withdrew/insufficient[2]	51	52	63	71	75	
**Stool markers**	Present/died[1]	160	72	138	132	143	2.5
	Missed visit/ withdrew/insufficient[2]	104	192	126	132	121	
	**TOTAL/marker**	**264**	**264**	**264**	**264**	**264**	
